# Effectiveness of registered nurses on patient outcomes in primary care: a systematic review

**DOI:** 10.1186/s12913-022-07866-x

**Published:** 2022-06-03

**Authors:** Julia Lukewich, Ruth Martin-Misener, Allison A. Norful, Marie-Eve Poitras, Denise Bryant-Lukosius, Shabnam Asghari, Emily Gard Marshall, Maria Mathews, Michelle Swab, Dana Ryan, Joan Tranmer

**Affiliations:** 1 Faculty of Nursing, Memorial University, 300 Prince Phillip Drive, St. John’s, NL A1B 3V Canada; 2 School of Nursing, Dalhousie University, 5869 University Ave. St, Halifax, NS B3H 4R2 Canada; 3 School of Nursing, Columbia University, 630 West 168th Street, New York, NY 10032 USA; 4 Département de Médecine de Famille Et Médecine d’urgence, Université de Sherbrooke, 2500 Boulevard de l’Université, Sherbrooke, QC J1K 2R1 Canada; 5 School of Nursing, McMaster University, 1280 Main St W, Hamilton, ON L8S 4L8 Canada; 6 Department of Family Medicine, Memorial University, 300 Prince Phillip Drive, St. John’s, NL A1B 3V6 Canada; 7 Department of Family Medicine Primary Care Research Unit, Dalhousie University, 1465 Brenton Street, Suite 402, Halifax, NS B3J 3T4 Canada; 8 Department of Family Medicine, Schulich School of Medicine & Dentistry, University of Western, 1151 Richmond Street, OntarioLondon, ON N6A 5C1 Canada; 9 Health Sciences Library, Faculty of Medicine, Memorial University, 300 Prince Phillip Drive, St. John’s, NL A1B 3V6 Canada; 10 School of Nursing, Queen’s University, 92 Barrie Street, Kingston, ON K7L 3N6 Canada

**Keywords:** Effectiveness, Primary healthcare, Registered nurse, Primary care nursing, Systematic review, Outcomes, Patient measures

## Abstract

**Background:**

Globally, registered nurses (RNs) are increasingly working in primary care interdisciplinary teams. Although existing literature provides some information about the contributions of RNs towards outcomes of care, further evidence on RN workforce contributions, specifically towards patient-level outcomes, is needed. This study synthesized evidence regarding the effectiveness of RNs on patient outcomes in primary care.

**Methods:**

A systematic review was conducted in accordance with Joanna Briggs Institute methodology. A comprehensive search of databases (CINAHL, MEDLINE Complete, PsycINFO, Embase) was performed using applicable subject headings and keywords. Additional literature was identified through grey literature searches (ProQuest Dissertations and Theses, MedNar, Google Scholar, websites, reference lists of included articles). Quantitative studies measuring the effectiveness of a RN-led intervention (i.e., any care/activity performed by a primary care RN) that reported related outcomes were included. Articles were screened independently by two researchers and assessed for bias using the Integrated Quality Criteria for Review of Multiple Study Designs tool. A narrative synthesis was undertaken due to the heterogeneity in study designs, RN-led interventions, and outcome measures across included studies.

**Results:**

Forty-six patient outcomes were identified across 23 studies. Outcomes were categorized in accordance with the PaRIS Conceptual Framework (patient-reported experience measures, patient-reported outcome measures, health behaviours) and an additional category added by the research team (biomarkers). Primary care RN-led interventions resulted in improvements within each outcome category, specifically with respect to weight loss, pelvic floor muscle strength and endurance, blood pressure and glycemic control, exercise self-efficacy, social activity, improved diet and physical activity levels, and reduced tobacco use. Patients reported high levels of satisfaction with RN-led care.

**Conclusions:**

This review provides evidence regarding the effectiveness of RNs on patient outcomes in primary care, specifically with respect to satisfaction, enablement, quality of life, self-efficacy, and improvements in health behaviours. Ongoing evaluation that accounts for primary care RNs’ unique scope of practice and emphasizes the patient experience is necessary to optimize the delivery of patient-centered primary care.

**Protocol registration ID:**

PROSPERO: International Prospective Register of Systematic Reviews. 2018. ID=CRD42 018090767.

**Supplementary Information:**

The online version contains supplementary material available at 10.1186/s12913-022-07866-x.

## Background

Primary care is the foundation of a highly functioning health care system and provides comprehensive, patient-centered care that considers the needs and experiences of the individual patient, their families, and the well-being of the broader community [1]. Primary care providers are the first contact and principal point of continuing care for patients within the health care system, and coordinate other specialist care and services that patients may need [1, 2]. The delivery of primary care occurs across varied settings but is most frequently provided in a clinic and, increasingly, by interprofessional teams that may consist of family physicians, registered nurses (RNs), nurse practitioners, pharmacists, and other health professionals. In primary care settings, RNs function as generalists and provide a broad range of patient services across the lifespan, including preventative screening, health education and promotion, chronic disease prevention and management, and acute episodic care [3–6]. Specifically, family physicians and RNs represent a key collaborative relationship within these teams, contributing to strengthened primary care delivery and improvements in the comprehensiveness, efficiency, and value of care for patients [7–9]. Internationally, nurses are increasingly embedded in primary care settings and are recognized as the most prominent non-physician contributor to primary care teams, although the scope and speed of implementation in this area differs across countries [10, 11]. Primary care nursing in Australia is the fastest growing employment sector, with 63% of general practices employing a primary care nurse (and 82% of this group representing RNs) [12, 13]. The World Health Organization’s report [14] on the state of the world’s nursing workforce emphasizes the need to strengthen the integration of RNs into primary care, as well as the need for further research to evaluate their impact. Global workforce data are unavailable given the variability in scope of practice and role terminology, and the lack of available information across countries. A recent review of the international literature identified that titles used to refer to RNs in primary care vary across countries [15]. For instance, titles for this role in Canada are ‘family practice nurse’ and ‘primary care nurse’, whereas in Australia, the United Kingdom, and Netherlands this title is known as ‘general practice nurse’ [15]. For the purpose of this manuscript, ‘primary care RN’ will be used throughout.

Most research in this area to date has focused on describing the roles and activities of primary care RNs. A systematic review conducted by Norful et al. [5] synthesized 18 studies from eight countries related to primary care RNs and identified assessment, monitoring, and follow-up of patients with chronic diseases as fundamental roles of the primary care RN. In contrast, there have been a number of reviews conducted on the effectiveness of nurse practitioners in primary care [16–18]. It is imperative that primary care RNs also begin to demonstrate their contributions to patient care within this setting. Research examining RN effectiveness has primarily been conducted within the acute care and long-term care settings and focused on staffing, role enactment, and work environment. Within these settings, there is substantial evidence demonstrating the positive effects of RN staffing on improving care and reducing adverse outcomes for hospitalized patients [24, 25].

Furthermore, select countries including Australia, Canada, New Zealand, and the United Kingdom have developed national standards of practice or competencies to define the scope and depth of practice for primary care RNs [4, 19–23]. National competencies for primary care RNs were recently published in Canada [7]. These competencies articulate the unique scope of practice and contributions to patient care for primary care RNs across six overarching domains, namely, (1) Professionalism, (2) Clinical Practice, (3) Communication, (4) Collaboration and Partnership, (5) Quality Assurance, Evaluation and Research, and (6) Leadership.

### Theoretical foundation

Determining effectiveness normally requires an examination of an intervention (e.g., primary care nursing) on a particular outcome. Incorporation of the patient perspective offers a more complete understanding of the challenges patients face within our healthcare system, especially those with long-term chronic diseases. Measuring the patient experience, which is a strong predictor of quality and value of care, should be done systematically [26]. The Organization for Economic Cooperation and Development (OECD) Patient Reported Indicator Surveys (PaRIS) Conceptual Framework was developed through a comprehensive process involving extensive international collaborations and provides a roadmap and survey tools (i.e., patient and provider questionnaires) to focus the evaluation of health care interventions on patient-reported metrics [27]. This framework provides a fuller evaluation of performance by complimenting other metrics (e.g., system/cost outcomes), while also focusing attention on the needs of the patient. The main domains of the framework include: patient reported experience measures (PREMs), patient reported outcome measures (PROMs), and health behaviours (e.g., physical activity, diet, tobacco use, alcohol use). Within primary care, the PaRIS Framework can serve as a guide for routine collection of these outcomes to facilitate quality improvement and patient-centered care [27]. A growing body of research in this area has adapted the use of this model to serve as an organizational and methodological framework. For example, multiple studies have used this framework as a method of investigating the suitability and feasibility of questionnaire and survey instruments when addressing patient perspectives [28, 29] or in the evaluation of health-related quality of life measures from the patient’s perspective [30]. A recently published systematic review that explored the opportunities and challenges of routine collection of PREMs and PROMs data for melanoma care within primary care settings found that these measures can address important care gaps and facilitate research and assessment [31]. Similarly, a study employing qualitative methods found that the use of patient-reported measures by practitioners enhanced patients’ ability to self-manage, communicate, engage, and reflect during consultations [32]. A recent environmental scan of the PROMs landscape was conducted within Canada and internationally, indicating a lack of standardized programs for routine collection and reporting of patient outcomes. Consequently, the need for enhanced PROMs information has been identified as an area of high priority [33].

### Purpose

Although existing literature provides some information about the contributions of primary care RNs towards outcomes of care, a systematic review synthesizing the effectiveness of the primary care RN workforce is needed. Prior to beginning the study, the Cochrane Database of Systematic Reviews, the Joanna Briggs Institute (JBI) Library of Systematic Reviews, and the Prospective Register of Systematic Reviews (PROSPERO) were searched and no existing registered protocols or previous systematic reviews on this topic were identified. Evaluating PREMs, PROMs, and health behaviours, as well as other patient-level outcomes, is necessary to accurately demonstrate the contribution of primary care RNs, hold them accountable for their care, and generate evidence to inform decisions and policies that impact their implementation and optimization [34, 35]. Therefore, the purpose of this systematic review is to summarize evidence examining primary care RNs’ impact on patient outcomes, including physiologic changes (via biomarkers), PREMs, PROMs, and health behaviours.

## Methods

### Design

A systematic review of effectiveness was conducted using JBI Systematic Review methodology [36] and findings were reported in accordance with the 2009 (and where possible, the 2021) Preferred Reporting Items for Systematic Reviews and Meta-Analysis (PRISMA) framework [37, 38]. Covidence software was used to manage and organize the literature [39] and enable a team approach for study and data review. The protocol for this systematic review is registered on PROSPERO (registration ID CRD42018090767). This paper presents a summary of findings from studies that report on patient outcomes, including biomarkers, PREMs, PROMs, and health behaviours. A full description of the methods and findings from studies that measured care delivery and system outcomes are reported in the companion paper *“Effectiveness of Registered Nurses on System Outcomes in Primary Care: A Systematic Review”* [40].

### Search strategy

The search strategy aimed to include both published and unpublished literature. Following a limited search in CINAHL and Medline that identified optimal search terms, two members of the research team performed a comprehensive search of relevant electronic databases (see Supplementary File 1). Grey literature was identified using ProQuest Dissertations and Theses, MedNar, Google Scholar, the websites of relevant nursing organizations (e.g., International Council of Nurses, Community Health Nurses of Canada), and reference lists of included articles. There were no location or publication date restrictions on search criteria. Studies published in any year up to and including the date of article retrieval (January, 2022) were considered. Ongoing searches for grey literature included studies with publication dates up to January, 2022.

### Inclusion criteria

Studies considered for inclusion reported on any quantitative study published in English with outcomes that directly measured, or were related to, an intervention attributable to a primary care RN. Only studies focused on RNs or equivalent (e.g., practice nurse, general nurse) [15] were included; if the RN designation was unclear or could not be determined based on the region of publication, the study was excluded. Studies that involved primary care RNs who underwent considerable advanced/focused training or those that exclusively examined structural variables were excluded. Full details regarding inclusion criteria are published in the companion paper [40].

### Screening

Reviewers included two study authors (DR and JL) and two trained research assistants (AR and OP). All identified titles and abstracts were screened independently by two reviewers for potential study eligibility. Two reviewers independently screened full-text articles for relevance, applying pre-established eligibility criteria. Any disagreements were resolved through discussion, or by a third reviewer.

### Risk of bias

The risk of bias and quality of each study was assessed using the *Integrated Quality Criteria for Review of Multiple Study Designs *(ICROMS) tool (see scoring matrix located in Supplementary File 2) [41]. All full-text articles that met eligibility criteria were appraised for quality by two independent reviewers. All studies that met inclusion/exclusion criteria also met the minimum ICROMS score to be included in the review.

### Data extraction and synthesis

All eligible full-text studies underwent data extraction using a tool pre-designed and tested by the research team and based on the Cochrane Public Health Group Data Extraction Template [42]. Data extracted from the articles included: country and year of publication, study aim and design, description of primary care setting, patient sample sizes and demographics, details of study intervention and primary care RN involvement/role, outcome measures used to evaluate these interventions, and study results. To address the broad range of terms and descriptors used across included studies (e.g., traditional care, standard care, basic support, care delivered by anyone other than a primary care RN) and to provide clarity in the presentation of our results, we refer to all control groups as “usual care” or the “comparator group”. Outcomes were grouped in accordance with the OECD PaRIS Conceptual Framework Classification [27] into one of three categories defined by this model (i.e., PREMS, PROMs, health behaviours), and an additional category added by the research team (i.e., biomarkers) (see Table 1). Biomarkers consist of outcomes related to changes in patient health status as measured by clinical assessment (e.g., hemoglobin A1c [HbA1c] values, blood pressure, body weight). PREMs are defined as patient experience indicators related to health care access, autonomy in care, and overall satisfaction with care received, and are often assessed through self-report questionnaires or population-based surveys [27]. These outcomes can be summarized further based on patient experiences surrounding access (e.g., first point of contact), comprehensiveness of care, self-management support, trust, and overall perceived quality of care. PROMs are described as outcomes relating to a patient’s self-reported physical, mental, and social health status and can be categorized as either generic or condition-specific and applied to a broad patient population [27]. Outcomes identified on this level can be further categorized into functional status (e.g., disability, physical, mental, social function), symptoms, and health-related quality of life. The remaining outcomes were categorized according to the health behaviors classification, which includes lifestyle behaviors and actions that can contribute to a patient’s overall health status (e.g., physical activity, smoking status, dietary intake) [27]. Due to the diversity of included designs, interventions, and outcomes across studies, narrative synthesis was used to present study findings.

**Table 1:** Classification of patient outcomes measured in each study based on the OECD PaRIS Conceptual Framework [27]

	Aubert et al., 1998	Aveyard et al., 2007	Bellary et al., 2008	Byers et al., 2018	Caldow et al., 2006	Cherkin et al., 1996	Coppell et al., 2017	Desborough et al., 2016	Faulkner et al., 2016	Gallagher et al., 1998	Halcomb, Davies, et al., 2015	Halcomb, Salamonson, et al., 2015	Harris et al., 2015	Harris et al., 2017	Iles et al., 2014	Karnon et al., 2013	Marshall et al., 2011	Moher et al., 2001	O'Neill et al., 2014	Pearson et al., 2003	Pine et al., 1997	Waterfield et al., 2021	Zwar et al., 2010
Biomarkers	✔		✔				✔						✔	✔		✔	✔		✔		✔	✔	
PREMs					✔	✔		✔	✔	✔	✔	✔					✔						✔
PROMs	✔					✔							✔	✔	✔		✔	✔		✔			
Health Behaviours		✔		✔		✔			✔				✔	✔			✔				✔		✔

## Results

**Fig. 1:**
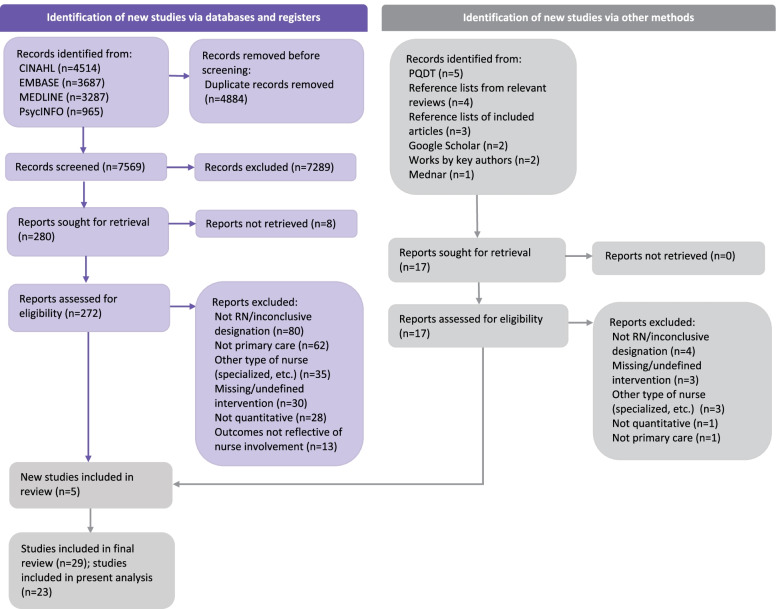
PRISMA Diagram of Literature Search. *This paper reports on studies that measured patient outcomes. Findings from studies that measured care delivery and system outcomes are reported in the companion paper "Effectiveness of registered nurses on system outcomes in primary care: a systematic review" [40]

Figure 1 presents a PRISMA diagram outlining the results of the literature search.

### Study characteristics

Of the 29 articles included in the final review, 23 reported on patient outcomes (included in the present analysis) [40]. Table 2 presents a detailed summary of the study characteristics for each of these articles. Studies were published between the years 1996–2021 and conducted in the United Kingdom (*n* = 9), United States (*n* = 6), Australia (*n* = 5), and New Zealand (*n* = 3). Study designs included randomized controlled trials (*n *= 9), observational (*n* = 8) (e.g., survey, secondary data analysis), cohort (*n* = 1), non-controlled (*n* = 2) and controlled before-after (*n* = 1), and two studies with mixed-methods designs that combined a non-controlled before-after with a non-randomized controlled trial (*n* = 1) or with an observational design (*n* = 1). Sample sizes ranged from 81–2850 patients. Quality scores, as assessed by the ICROMS tool, varied between studies. Four studies were scored at the minimum threshold for their study design [46, 56, 61, 65], six studies scored 1–2 points above threshold [44, 45, 48, 49, 53, 57], and thirteen studies exceeded the minimum cut-off score by 3 or more points [43, 47, 50–52, 54, 55, 58–60, 62–64].

**Table 2:** Literature review table of study characteristics (*n* = 23)

Author, Year, Country	Aim	Design	Sample	Intervention and RN Involvement	Primary Care Setting Type	ICROMS Quality Appraisal Score [1]
Aubert et al., 1998 [43] USA	To compare diabetes control in patients receiving nurse case management and patients receiving usual diabetes management in a primary care setting	Randomized controlled trial	Prudential HealthCare health maintenance organization members with diabetes (*n* = 138 patients were randomized; *n *= 100 provided 12-month follow-up data)	Nurse case management for patient diabetes control (diabetes management delivered by a RN case manager) v. usual diabetes care (control) One RN provided the intervention for this study; RN had 14 years of clinical experience and was a certified diabetes educator RN provided intervention with support - met at least biweekly with the family medicine physician and the endocrinologist to review patient progress and medication adjustments. RN was trained in the delivery of care while primary care providers oversaw clinical decisions	2 primary care clinics within a group-model health maintenance organization in Jacksonville, Florida	25
Aveyard et al., 2007 [53] UK	To examine whether weekly behavioral support increased smoking quit rate relative to basic support; and to assess whether primary care nurses can deliver effective behavioral support	Randomized controlled trial	Adults who smoked ≥ 10 cigarettes per day (*n* = 925) were recruited between July 2002 - March 2005	Smoking cessation support provided by a PN (weekly behavioural support [one additional visit and two additional telephone calls]) v. basic, less frequent support provided by a PN (control) Number of PNs and additional characteristics were not indicated PN provided intervention alone. All PNs were provided with mentoring and training on the application of smoking cessation support, as well as information on the use of nicotine replacement therapies	26 general practices in two urban counties in the UK	23
Bellary et al., 2008 [44] UK	To investigate the effectiveness of a culturally sensitive, enhanced care package for improvement of cardiovascular risk factors in patients of South Asian origin with type 2 diabetes	Cluster randomized controlled trial	Adult patients of South Asian origin with type 2 diabetes (*n* = 1486)	Enhanced management care for type 2 diabetes tailored to the needs of the South Asian community (enhanced care [additional time with PN + support with link worker and diabetes-specialist nurse] v. standard care/control [routine PN-led diabetes clinics guided by prescribing algorithm]) Number of PNs not indicated; all were formally trained in diabetes management PN provided intervention with support of diabetes nurse specialist, link worker, and physician. PNs worked with primary care physicians to implement the protocol and encourage appropriate prescribing, provide patient education, and achieve health targets	21 inner-city practices in 2 cities in the UK with a high-population of South Asian patients. Patients were randomly allotted to the intervention or the control group between March 2004 - April 2005	24
Byers et al., 2018 [54] USA	To compare smoking cessation rates between nurse-led and physician-led preventative/wellness visits	Observational; retrospective secondary analysis of a de-identified electronic medical record data set	Medicare beneficiaries who received wellness visits or non-Medicare patients who received MD-led annual physicals and identified as smokers (*n* = 218) between January 2011 - December 2015	Nurse-led wellness visits focused on smoking cessation carried out by RNs v. same intervention carried out by GPs Nurses in the RN-led group were non-advanced practice RNs. Due to limited resources and competing clinical demands, efforts to implement Medicare annual wellness visits occurred gradually and included RNs in 4 of the 6 clinics RN provided intervention alone, carrying out point-of-care screening and various other preventative wellness activities	Network of 6 primary care clinics in Arkansas, USA	20
Caldow et al., 2006 [64] UK	To assess patients’ satisfaction, attitudes, and preferences regarding PN v. doctor consultations for minor illness as first-line contact	Observational; survey and telephone interviews	Large random sample of registered patients over 18 years of age (*n* = 2949 questionnaires were mailed out; *n* = 1343 [45.5%] were returned completed)	National survey of patient satisfaction, attitudes, and preferences regarding PN care v. doctor consultation Number of PNs and additional characteristics were not indicated. Data obtained from postal questionnaire survey including discreet choice experiment, followed by telephone interviews Practices were scored and ranked according to the degree of extended nursing role; the 20 most and 20 least extended practices according to the criteria were invited to participate Organizational-level involvement; practices had PNs with varying roles (traditional and extended) and patients were surveyed about their interactions and attitudes/preferences	433 general practices, including traditional and extended PN roles in Scotland	21
Cherkin et al., 1996 [57] USA	To evaluate the impact of a proactive and patient-centered educational intervention for low back involving a nurse-intervention group in comparison with two lower impact treatment models	Randomized controlled trial	Patients aged 20–69 years of age visiting the clinic for back pain, low back pain, hip pain, or sciatica (*n* = 294) were randomly allocated to one of 3 groups; *n* = 286 provided complete follow-up data	Educational intervention for back pain carried out by a RN (usual care) v. usual care + educational booklet (intervention arm 1) v. usual care + session with RN + educational booklet (intervention arm 2); outcomes assessed at 1, 3, 7, and 52 weeks Study involved 6 female RNs with at least 20 years of clinical experience. Study RNs received 9 h of training on the management of back pain RN provided intervention alone. The intervention involved a 15–20-min educational session, including the booklet and a follow-up telephone call 1–3 days later	Suburban primary care clinic in western Washington state, belonging to a staff model Health Maintenance Organization	24
Coppell et al., 2017 [50] New Zealand	To examine the implementation and feasibility of a six-month multilevel primary care nurse-led prediabetes lifestyle intervention compared with current practice for patients with prediabetes	Pragmatic, non-randomized controlled before-after; convergent mixed methods design	Non-pregnant adults aged ≤ 70 years with newly diagnosed prediabetes, a BMI above 25, not prescribed Metformin, and able to communicate in English were sent a study invitation letter between August 2014 -April 2015. One-hundred fifty-seven patients were enrolled and *n* = 133 patients were retained at the six-month follow-up	Multi-level primary care nurse-led prediabetes lifestyle intervention involving a structured dietary intervention tool v. usual care (control) Study involved 11 RNs and community nurses. Additional characteristics were not indicated RN provided intervention with support of dietician and liaison nurse. RNs delivered the clinic portion of the intervention (30-min dietary session and evaluation using a validated dietary measurement tool at baseline, 2–3 weeks, 3 months, and 6 months) while community nurses carried out the group education sessions outside of the clinic	4 intervention general practices and 4 control general practices located in 2 neighboring New Zealand cities; all practices employed a primary care nurse	26
Desborough et al., 2016 [62] Australia	To examine the relationships between specific general practice characteristics, nurse consultation characteristics, and patient satisfaction and enablement	Observational; cross-sectional survey	Patients in general practice who had consulted with a nurse, regardless of health condition, and were 16 years or older, or 5 years or younger (*n* = 678) between September 2013 - March 2014	Nursing care in general practice based on specific practice characteristics and nurse consultation characteristics (measured by patient surveys and interviews with nurses, patients, and practice managers) Study involved 47 baccalaureate-prepared RNs and 3 diploma-prepared enrolled nurses across all practices (average of 2.5 RNs per practice) with a mean of 3 years of experience RN provided intervention alone. The majority of consultations were for clinical care, preventative health care, and chronic disease management	21 general practice locations in an Australian capital territory, with an average ratio of 3–4 GPs: 1 nurse per clinic	22
Faulkner et al., 2016 [55] UK	To compare differences in smoking cessation treatment delivered by PNs or HCAs on short and long-term abstinence rates from smoking	Cohort study using longitudinal data from a previously conducted randomized controlled trial	Current smokers aged 18–75 years who are fluent in English, not enrolled in another formal smoking cessation study or program, and not using smoking cessation medications (*n* = 602)	Smoking cessation support provided by PNs v. HCAs to compare and assess effects on short and long-term smoking abstinence rates on patients Number of PNs and additional characteristics were not indicated PNs provided intervention alone (and were compared to same intervention provided by HCAs). Patients in both groups received an initial consultation, followed by a program-generated cessation advise report tailored to the smoker and a 3-month program of tailored text messages sent to their mobile phone	32 general practices in East England; 8 of which were in the top 50% of deprived small geographical areas in England	21
Gallagher et al., 1998 [65] UK	To determine the impact of telephone triage, conducted by a PN, on the management of same day consultations in a general practice	Observational (cross-sectional) and uncontrolled before-after using prospective telephone and practice consultation data + patient postal questionnaire	All patients in practice (*n* = 1250 consultations with diagnosis), in which consultations were recorded between August - October 1995	Nurse operated telephone consultations/triage There was a total of 4 PNs working in the practice; the telephone consultation/triage service was managed by a single nurse who had 15 years of experience and was familiar with managing acute illnesses and conducting telephone consultations PN provided intervention with support of physician and receptionist. Patients who telephoned requesting to see a doctor on the same day were put through to the PN, where they would manage the patient’s problem over the phone or arrange for a same-day appointment with either themselves or the GP	Individual general practice in an urban city in England that contains physicians, PNs, and admin staff	16; 22*
Halcomb, Davies, & Salamonson, 2015 [63] New Zealand	To understand the relationship between consumer demographics and their satisfaction with PN services	Observational; survey	Patients with sufficient fluency in English to complete the survey form and provide consent (*n* = 1505) were recruited through email invitation from December 2010 - December 2011	PN-led care in general practice as assessed by a 64-item self-report survey tool completed by patients Number of PNs not indicated, however each participating practice employed between 1–11. All participating nurses were female and had an average of 22 years of experience, a mean age of 49 years, and worked between 8–44 h/week PN provided intervention alone by performing a range of services within primary care nursing scope of practice- vaccination, blood pressure measurement, cardiovascular assessments, treatment of minor illnesses/injuries, cervical smears and sexual health check-ups, tissue collection, lung function tests, etc	20 general practices in New Zealand, representing a mixture of urban and rural locations	21
Halcomb, Salamonson, & Cook, 2015 [45] Australia	To evaluate consumer satisfaction and comfort with chronic disease management by nurses in general practice	Observational; survey	A convenience sample of all patients in practice (*n* = 81) who received services from a participating PN	Chronic disease services delivered by PNs in general practice, as measured by a 33-item survey tool Number of PNs and additional characteristics were not indicated PN provided intervention alone; after services were delivered, patients were provided with an information package containing a survey to evaluate their encounter	8 general practices that contained GPs and PNs working collaboratively in New South Wales, Australia	17
Harris et al., 2015 [59] UK	To determine whether a primary care nurse-delivered complex intervention increased objectively measured step-counts and moderate to vigorous physical activity when compared to usual care	Cluster randomized controlled trial	60–75-year-olds who could walk outside and had no contraindications to increasing physical activity (*n* = 298 patients from* n* = 250 households) were recruited between 2011 - 2012 from a random sample of eligible households	Individually-tailored PN consultations centered around physical activity (four physical activity consultations with nurse) v. usual care (no trial contacts other than for data collection at baseline, 3 months, and 12 months) (control) Number of PNs and additional characteristics were not indicated PN provided intervention alone; physical activity consultations incorporated behavioural change techniques, step-count and accelerometer feedback, and an individual physical activity plan	3 general practices located in Oxfordshire and Berkshire, UK	28
Harris et al., 2017 [60] UK	To evaluate and compare the effectiveness of pedometer-based and nurse-supported interventions v. postal delivery intervention or usual care on objectively measured physical activity in predominantly inactive primary care patients	Cluster randomized controlled trial	A random sample of 45–75-year-olds without contraindications to increasing moderate to vigorous physical activity (*n* = 956 with at least one follow-up) were sent postal invitations between September 2012 - October 2013	Nurse-supported individually-tailored physical activity consultations as measured by patient pedometer activity (nurse-supported pedometer intervention [arm 1]) v. postal pedometer intervention [arm 2] v. usual care [control]) Number of PNs and additional characteristics were not indicated PN provided intervention alone; nurse-supported intervention group involved a pedometer, patient handbook, physical activity diary, and three individually tailored PN consultations offered at 1, 5, and 9 weeks	7 general family practices with an ethnically and socioeconomically diverse population in South London	26
Iles et al., 2014 [46] Australia	To determine the economic feasibility of using a PN-led care model of chronic disease management in Australian general practices in comparison to GP-led care	Randomized controlled trial; cost-analysis	Patients > 18 years of age with one or more stable chronic diseases (type 2 diabetes, ischemic heart disease, hypertension) (*n *= 254)	PN-led care model of chronic disease management (*n *=120) v. GP-led (usual care) care model (*n* =134) There were 2 PNs and 1–4 GPs involved in each practice over the 2-year study period PN provided intervention alone, working within their scope of practice and from protocols, rather than under supervision of GP; if patients in the PN-led group became unstable, they could be referred back to the GP-led group until their health re-stabilized	3 general practices (urban, regional, rural)	22
Karnon et al., 2013 [47] Australia	To conduct a risk adjusted cost-effectiveness analysis of alternative applied models of primary health care for management of obese adult patients based on level of PN involvement (high-level PN practice v. low-level PN practice v. physician-only model)	Observational; risk-adjusted cost-effectiveness analysis	Patients with BMI < 30 prior to October 1, 2009, had at least three visits within the last 2 years, at least two recorded measures of BMI, and aged 18–75 years (*n* = 383 patients were recruited, *n* = 208 were excluded, *n *= 150 patients included in the analysis) who gave consent for researchers to access their medical data	PN involvement in the provision of clinical-based obesity care. Models of care classification were based on percentage of time spent on clinical activities: high-level model (*n* = 4), low-level model (*n* = 6), physician-only model (*n* = 5; due to low number of eligible patients in the physician-only model, data were not presented) Number of PNs were not indicated, although results indicate that high level practices had a non-significantly higher number of FTE PNs than low level practices (0.35 compared to 0.25 for low level practices, *p *= 0.34); PNs had varying scopes of practice in clinics, which was informed by survey responses which assessed their clinical-based activities No specific nurse intervention; study examined nursing care related to obesity in general (e.g., education, self-management advice, monitoring clinical progress, assessing treatment adherence)	15 of 66 general practices within the Adelaide Northern Division of General Practice with varying levels of PN involvement	22
Marshall et al., 2011 [51] New Zealand	To assess patients’ experiences and opinions of the Nurse-Led Healthy Lifestyle Clinic Project as well as recorded clinical outcomes, and to assess how successfully the clinics engaged the target populations	Observational; clinical outcome data and cross-sectional surveys	Patients with a specifically diagnosed condition relevant to the nurse-led lifestyle clinics (diabetes, smoking cessation, women’s health, cardiovascular, respiratory, diet/nutrition) (*n* = 2850)	Nurse-led healthy habits lifestyle clinics for patients with or at risk of chronic disease within targeted populations with known health inequalities 115 RNs in total participated; in each clinic the nurses had their own patient caseload. Clinical outcome data were obtained from individuals who participated in the clinics (*n* = 2850) and patient satisfaction surveys (*n* = 424) RN provided intervention alone, however, in some cases patients were referred to other professionals when warranted. RNs delivered care using a holistic health approach defined by the patients’ needs. Clinical outcome data was collected on the first and last day of clinic attendance	17 practices (3 Hauora, 2 community, and 12 general practices) served by the Primary Healthcare Organization	20
Moher et al., 2001 [52] UK	To assess the effectiveness of three different methods for improving the secondary prevention of coronary heart disease in primary care (audit and feedback; recall to a GP; recall to a nurse clinic)	Pragmatic, unblinded, cluster randomized controlled trial comparing three intervention arms	Patients aged 55–75 years with established coronary heart disease (*n* = 1906) as identified by computer and paper health records were recruited from 1997 - 1999	Secondary prevention care of patients with coronary heart disease delivered at three levels (i.e., audit and feedback; GP recall; nurse recall) Number of PNs in study unknown- all practices employed at least 1 PN; additional characteristics not identified PN provided intervention with support of the trial’s nurse facilitator, who gave ongoing support to the practices in setting up a recall system for review of patients with coronary heart disease. The nurse recall and GP recall groups employed the same intervention	21 general practices in Warwickshire that employed PNs, but were not already running nurse-led clinics	26
O’Neill et al., 2014 [48] USA	To assess expanded CPS and RN roles by comparing blood pressure case management between CPS and physician-directed RN care in patients with poorly controlled hypertension	Observational; non-randomized, retrospective comparison of a natural experiment	Patients that had face-to-face or telephone appointments with a RN case manager for poorly controlled hypertension with either physician-directed or CPS-directed clinical decision making at the index encounter (*n *= 126)	Patient hypertension care delivered by CPS-directed RN case management as an alternative to physician-directed RN case management Number of RNs and additional characteristics were not indicated RN provided intervention with support of either CPS or physician; RNs assessed patients independently and presented the case to either a CPS or a physician, if the hypertension continued to be poorly controlled. The RN communicated any changes in the plan to the patient	A large Midwestern Veteran’s Affairs Medical Center that utilizes team-based care	18
Pearson et al., 2003 [61] USA	To apply the principles from the Kaiser Permanente model for depression treatment towards the development and implementation of a primary care PN telecare program	Uncontrolled before-after	Patients aged 21–64 years, diagnosed with major depressive disorder, depressive disorder NOS with severe symptoms, or dysthymic disorder, were experiencing a first or new episode of depression, and were prescribed an SSRI (*n* = 177 patients enrolled; *n* = 102 analyzed at six-month follow-up)	Nurse telecare case management program based on the principles from the Kaiser Permanente model for patients with diagnosed depression Study consisted of 12 RNs and 2 LPNs involved in the telephone follow-up portion; additional characteristics not identified Organizational-level intervention; providers consisted of 39 physicians, 6 NPs and 5 physician assistants. Telephone follow-up was provided by RNs alone, however, they could consult with a supervising psychiatrist on an as-needed basis	13 primary care practices in Maine’s urban centers of Augusta, Bangor, Lewiston, and Portland	22
Pine et al., 1997 [49] USA	To evaluate the effect of a nurse-based intervention for patients with high total cholesterol levels in a community practice	Non-controlled before-and-after clinical trial (pre-post study) followed by a non-randomized controlled trial (matching study)	One hundred twenty-three patients agreed to participate. Forty-one were excluded from the final analysis. The final sample consisted of *n* = 82 white patients with total cholesterol higher than 6.21 mmol/L	Counseling provided by nurses to patients diagnosed with hypercholesterolemia using the Eating Pattern Assessment Tool and handouts with food advice Study involved 2 RNs; additional characteristics not identified RN provided intervention alone; in the pre-post study, RNs provided 5 counseling visits (1 month after referral, and at 3, 5, 7, and 12 months) to patients, which were focused on nutritional education and physical activity. In the follow-up matching study, intervention patients who attended 2 or more counseling sessions were matched with other patients in the practice	Large multi-specialty group suburban primary care practice in Minneapolis	23; 24*
Waterfield et al., 2021 [58] UK	To determine whether primary care nurses with no prior experience can, after training, provide effective supervised PFMT, when compared to PFMT given by a urogynaecology nurse specialist and that of usual care	Randomized controlled trial	Sample consisted of 337 asymptomatic women with weak pelvic floor muscles (Modified Oxford Score 2 or less) in a randomly sampled survey. Two hundred forty women aged 19 - 76 (median 49) years were recruited	PFMT delivered to patients with weak pelvic floor muscles at three levels: primary care nurse-delivered training (arm 1) v. urogynaecology nurse specialist training (arm 2) v. usual care (no training) Number of primary care nurses involved and additional characteristics were not indicated; at least one primary care nurse from each practice participated Primary care nurse provided intervention alone; the primary care nurse intervention group were provided training materials related to pelvic floor assessment and techniques involved in teaching PFMT. Primary care nurses taught patients a PFMT regimen to perform 3–6 times per day for 3 months and used a perineometer to assess pelvic floor strength at baseline and 3 months	11 primary care/general practices, covering urban and rural settings in South West England	27
Zwar et al., 2010 [56] Australia	To examine the impact of PN-delivered behavioral support on smoking cessation rates as well as the feasibility and acceptability of this model to patients, PNs, and GPs	Non controlled pre- and post-study using mixed methods	A convenience sample of smokers (*n* = 498 initial; *n* = 378 at 6-month follow-up) were recruited during nurse appointment in general practice	Nurse-delivered smoking cessation counseling Study involved 31 PNs and all practices included in study employed at least 1 PN; additional characteristics not identified PNs took a leading role in providing counseling but were supported by the GPs from participating practices; GPs identified smokers interested in quitting and referred them to the PN for a series of weekly counseling visits of approximately 30-min duration over 4 weeks	19 general practices in South West Sydney and a nearby rural area, representing 2 Divisions of General Practice	22

*Mixed methods study consisting of multiple designs; separate ICROMS quality appraisal scores were generated for each study type; *RN *registered nurse, *PN *practice nurse, *MD *medical doctor, *BMI *body mass index, *FTE* full-time equivalent, *HCA *health care assistant, *GP *general practitioner, *CPS *clinical pharmacy specialist, *NP *nurse practitioner, *NOS *not otherwise specified, *SSRI *selective serotonin reuptake inhibitor; *LPN *licensed practical nurse, *PFMT *pelvic floor muscle training

### Overview of RN interventions

The nature of interventions examined in this review differed across studies. The most common interventions were related to chronic disease prevention and management, specifically, case management or targeted chronic disease management care (e.g., diabetes, obesity, hypertension, hypocholesteremia) (*n* = 7) [43–49] and primary and secondary preventative care for patients at risk of chronic disease (e.g. prediabetes, coronary heart disease) (*n* = 3) [50–52]. Other studies examined primary care RN-delivered smoking cessation support (*n* = 4) [53–56], back pain education and management [57], pelvic floor muscle training [58], consultations aimed at increasing patient physical activity levels [59, 60], and a telecare program for patients with diagnosed depression [61]. Four studies examined the impact of RN care in general (at an organizational-level); three of which focused on consultations with patients in general practice [62–64] and another which examined the impacts of a nurse-operated telephone consultation/triage service [65].

In thirteen studies, primary care RNs carried out the intervention independently without the support of other staff/providers [45, 46, 49, 51, 53–55, 57–60, 62, 63], and in 10 studies, they carried out the intervention interdependently, in collaboration with health care providers (e.g., physicians, clinical pharmacy specialists [CPS], dieticians) or members of the research team (e.g., trial nurse facilitator) [43, 44, 47, 48, 50, 52, 56, 61, 64, 65]. Three of these 10 studies involved evaluating RNs at the general practice-level and therefore are assumed to be evaluating an interdependent role involving support of other health care providers [47, 61, 64]. The presence and type of comparator group also differed across study designs. Specifically, five of the included studies compared a nurse-led intervention to the same intervention led by other health care providers [46, 52, 54, 55, 58]. Other studies compared nurse-led interventions with that of ‘usual care’ not associated with nurse involvement (*n* = 4) [43, 50, 57, 60], or with ‘usual care’ that was associated with reduced or alternative levels of nurse involvement (*n* = 5) [44, 49, 53, 56, 59]. The remaining studies examined the effectiveness of a primary care RN-delivered intervention on specific outcomes of care using an observational or before-after design (*n *= 5) [48, 51, 61, 62, 65], or did not contain a specific intervention, but rather, examined the impact of varying roles and practice characteristics of the primary care RN in general practice (*n* = 4) [45, 47, 63, 64].

### Overview of outcomes

A total of 46 patient outcomes were identified across included studies (Table 3). Physiologic disease control outcomes, which were measured via biomarkers, included quality of care for diabetes (e.g., HbA1c, fasting blood glucose) [43, 44, 50, 51], obesity (e.g., body mass index [BMI], waist circumference) [44, 47, 50, 51, 59, 60], pelvic floor strength and endurance [58], hypercholesterolemia (e.g., total cholesterol) [49], and hypertension (e.g., blood pressure) [43, 44, 48, 50, 51]. Patient experience outcomes identified under the PREMs category included patient satisfaction with access to care (RN versus physician as first point of contact) [64], quality of self-management support (e.g., smoking cessation counseling, chronic disease services) [56, 62], comfort/trust with primary care RN roles [45], and overall satisfaction or perceived quality of care with provider consultations, treatment, or advice/support received [45, 51, 55, 57, 63, 65]. Patient reported outcomes identified within the PROMs category consisted of physical and social functional status [43, 57], level of disability (e.g., activity levels, bed rest, work loss) [57, 61], changes in self-reported anxiety, depression, or pain [59–61], adverse health events (e.g., falls, fractures, severe hypoglycemia) [43, 59, 60], and health-related qualify of life (e.g., physical activity, social activity) [43, 46, 51, 52, 60]. Lastly, outcomes grouped under the health behaviors classification included reduction and/or cessation of tobacco use [51, 53–56], changes to physical activity (e.g., level of aerobic exercise, daily step count) [51, 57, 59, 60], and improvements in dietary intake [49].

**Table 3:** Literature Review Table – Description of Patient Outcomes and Study Results

Author, Year, Country	Description of Outcome	Results
**Biomarkers**		
Aubert et al., 1998 [43] USA	Changes in HbA1c value and other clinical markers related to diabetes management (fasting blood glucose, medication type and dose, body weight, blood pressure, lipid levels) after 12 months	The intervention group had a greater decrease in HbA1c values than did the usual care group. The average change in HbA1c value was -1.7 percentage points in the intervention group and -0.6 percentage points in the usual care group (difference -1.1, 95% CI: -1.62 to 0.58; *p* < 0.001). Patients in the intervention group had a greater decrease in fasting blood glucose than the usual care group (-48.3 mg/dL v. -14.5 mg/dL; difference -33.8, 95% CI: -56.12 to 11.48; *p* = 0.003); however, other measures were not significant The results show that a RN case manager, in association with primary care physicians and an endocrinologist, can help improve glycemic control in diabetic patients in a group-model health maintenance organization
Bellary et al., 2008 [44] UK	Changes in type 2 diabetes health markers (blood pressure, total cholesterol, HbA1c) after 2 years Changes in waist circumference, BMI, microalbuminuria, plasma creatinine, Framingham CHD risk after 2 years	The study produced only modest clinical outcomes when comparing the two groups in diastolic blood pressure (-1.91, 95% CI: -2.88 to -0.94 mm Hg; *p* = 0.0001) and mean arterial pressure (1.36, 95% CI: -2.49 to -0.23 mm Hg; *p *= 0.018); other outcomes (total cholesterol, systolic blood pressure, HbA1c) were not significant across the two groups. Across both arms of the study over the 2-year period, systolic and diastolic blood pressure decreased significantly and there was a small, but non-significant, reduction in HbA1c (-0.04%, 95% CI: -0.04 to -0.13; *p* = 0.29). There were no significant differences between groups for waist circumference, microalbuminuria, plasma creatinine or CHD risk score. BMI was significantly increased in the intervention group (*p* < 0.0001) Evidence suggests that intensive PN-led management can improve outcomes in type 2 diabetes, although this requires further development
Coppell et al., 2017 [50] New Zealand	Between-group changes to diabetes health markers (weight, HbA1c, waist circumference, BMI, blood pressure, lipids, urate, liver enzymes) after 6 months	The intervention group lost a mean 1.3 kg, while the control group gained 0.8 kg (2.2 kg difference; *p* < 0.001). Mean HbA1c, BMI, and waist circumference decreased in the intervention group and increased in the control group at 6 months, but differences were not statistically significant after 2 years. Implementation fidelity was high and the intervention was considered feasible to implement in busy general practice settings
Harris et al., 2015 [59] UK	Changes in patient BMI and fat mass at 3-month follow-up	There were no between-group differences in change to BMI (0.001 kg/m^2^, 95% CI: -0.17 to 0.18, *p* = 0.98) or fat mass (0.39 kg, 95% CI: -0.85 to 0.07; *p* = 0.10) at 3 months
Harris et al., 2017 [60] UK	Changes in patient fat mass, BMI and waist circumference at 3- and 12-month follow-up	Fat mass was slightly reduced at 12 months in both intervention groups, but these differences did not differ significantly when the nurse group was compared to both postal intervention (*p* = 0.54) and usual care (*p* = 0.30). There was no change in BMI or waist circumference
Karnon et al., 2013 [47] Australia	Weight loss as defined by changes in BMI and weight, as well as reduction of obesity-related complications	Relative to low level involvement of practice nurses in the provision of clinical-based activities to obese patients, high level involvement was associated with significantly larger mean reductions in BMI (mean difference -1.10, CI: -0.45 to -1.75; *p* = 0.001) after 1 year, and non-significant improvements with respect to patients losing any, 5 and 10% of their baseline weight (*p* = 0.259)
Marshall et al., 2011 [51] New Zealand	Changes to blood pressure, weight, BMI, HbA1c, waist circumference, and cardiovascular risk between patient’s first and last visit	No significant changes in average blood pressure, weight, BMI, HbA1c, waist circumference and cardiovascular risk assessment were detected between baseline and follow-up visits
O’Neill et al., 2014 [48] USA	Changes in blood pressure between index and next consecutive visit	Patients receiving CPS-directed RN case management had greater decreases in systolic blood pressure (-14 mm Hg) than those receiving physician-directed RN management (-10 mm Hg) (*p* = 0.04). After adjusting for time between visits, blood pressure, and prior stroke, there was no significant effect for provider type on systolic blood pressure change (*p*=0.24). There were no significant changes in diastolic blood pressure between groups. CPS-directed and physician-directed RN case management for hypertension demonstrated similar effects on blood pressure reduction, supporting an expanded role for CPS-RN teams
Pine et al., 1997 [49] USA	Changes in total cholesterol levels from first to final nurse visit (pre-post study)	Mean total cholesterol level decreased by 0.29 mmol/L (11.2 mg/dL) (4.3%) from the physician visit to the first nurse visit (*p* < 0.001) and 0.14 mmol/L (5.4 mg/dL) (2.1%) from the first nurse visit to the final nurse visit (*p* = 0.4).
	Differences in total cholesterol levels between intervention and comparison groups (matching study)	The mean total cholesterol level of all patients improved significantly (*p* = 0.002). However, the improvement in intervention patients was no better than that of comparison patients
Waterfield et al., 2021 [58] UK	Strength of pelvic floor muscle contraction	After 3 months, there was an increase in strength in both intervention groups compared with controls, with a median difference of 3.0 cmH_2_0 higher for the primary care nurse group compared to the control group (95% CI: 0.3 to 6.0;* p* = 0.02), and 4.3 cmH_2_0 for the urogynecology specialist group compared to the control group (95% CI: 1.0 to 7.3; *p* < 0.01). There was no difference between the primary care nurse and urogynecology nurse specialist groups (1.3; 95% CI: -2.0 to 4.7; *p* = 0.70)
	Endurance of pelvic floor muscle contraction	There was an overall significant difference in endurance over the three groups at the end of the study (*p* < 0.001). Endurance of contraction for both of the intervention groups increased, while there was a slight decline for the controls from baseline endurance levels. Both the primary care nurse group and the urogynecology nurse specialist group had a significant increase in endurance compared to the control group at 3 months (*p* = 0.009 and *p* = 0.008, respectively)
**Patient-Reported Experience Measures (PREMs)**		
Caldow et al., 2006 [64] UK	Patient satisfaction with, opinion of, and preference for PN v. doctor consultation in primary care derived from questionnaire responses	Women, younger people, and those who had a lower level of education were significantly less satisfied with the time spent if they had seen a GP compared with a PN (*p* < 0.05). Patients reported more satisfaction in this area in practices where the PN had an extended role (*p *< 0.001) Women and younger people had a significantly higher positive attitude towards, and perception of, PNs than did men and older people, respectively (*p* < 0001), and thought that a PN would know their family history as well as a GP would (*p* < 0.05). Younger and less well educated people perceived that a PN would know their medical condition (*p* < 0.001) as well as a GP would. The main perceived differences between GPs and PNs was academic ability and qualifications. This suggests that if PNs take on more roles that were previously only within GP scope of practice, patients would accept them, particularly if they receive information about nurse capabilities
Cherkin et al., 1996 [57] USA	Patient satisfaction evaluated based on 5 dimensions of subjects’ perceptions: perceived knowledge, worry, control, symptoms, and evaluation of care	The nurse intervention resulted in higher patient satisfaction than usual care (*p* < 0.05) and higher perceived knowledge (*p* < 0.001). There were no significant differences among the three groups in worry or symptoms at any follow-up interval and differences in knowledge were no longer significant at the 52-week follow-up
Desborough et al., 2016 [62] Australia	Patient scores on the Patient Enablement and Satisfaction Survey regarding nurse-led consultations	The median total satisfaction score was 63, indicating that patients were either satisfied or very satisfied with nursing care. The median total patient enablement score was 2.25, indicating enablement levels of the same or less than the average, or that the questions were not applicable. Patients who had longer consultations were more satisfied (OR = 2.50, 95% CI: 1.43 to 4.35; *p* < 0.01) and more enabled (OR = 2.55, 95% CI: 1.45 to 4.50; *p* < 0.01) than those who had shorter consultations. Patients who had continuity of care (6 or more appointments) with the same nurse were more satisfied (OR = 2.31, 95% CI: 1.33 to 4.00; *p* = 0.01). Patients who attended practices where nurses worked with broad scopes of practice and high levels of autonomy were more satisfied (OR = 1.76, 95% CI: 1.09 to 2.82; *p* = 0.04) and more enabled (OR = 2.56, 95% CI: 1.40 to 4.68; *p* < 0.01). Patients who received care for the management of chronic conditions (OR = 2.64, 95% CI: 1.32 to 5.30; *p* < 0.01) were more enabled than those receiving preventive health care These results provide evidence of the importance of continuity of nursing care, adequate consultation time, and broad scopes of nursing practice and autonomy for patient satisfaction and enablement
Faulkner et al., 2016 [55] UK	Patient satisfaction with initial consultations (how clear they found the advice received on pharmacotherapies, the usefulness of cessation advice received, and satisfaction with consultation as a whole) as assessed by self-report questionnaire	Patients in both groups gave positive evaluations of the support they received; 93.2% of patients who saw HCAs and 91.2% who saw nurses said they were ‘happy’ or ‘extremely happy’ with the consultations, and 89.5% and 84.5% of patients who saw HCAs and nurses, respectively, reported finding the advice they received ‘useful’ or ‘extremely useful’. There were no statistically significant differences in any aspect of patient satisfaction by provider type
Gallagher et al., 1998 [65] UK	Patient satisfaction with nurse-led telephone advice as measured by a postal questionnaire	Most (*n* = 154; 88%) patients were very or fairly satisfied with nurse telephone advice. Only *n* = 10 (6%) were fairly or very dissatisfied
Halcomb, Davies, & Salamonson, 2015 [63] New Zealand	Patient perceptions of PNs based on responses to a 64-item self-report survey tool containing the General Practice Nurse Satisfaction scale	Participants over 60 years and those of European descent were significantly less satisfied with the PN (*p* = 0.001); however, controlling for these characteristics, participants who had made < 4 visits to the PN were 1.34 times (95% CI: 1.06–1.70) more satisfied than the comparison group. The study also revealed a high level of satisfaction with PNs overall, with increased satisfaction associated with an increased number of visits Findings suggests that age, ethnicity and employment status were significant predictors of satisfaction levels, and that greater continuity with the PN (i.e., number of visits) strongly influences patient satisfaction with nursing services
Halcomb, Salamonson, & Cook, 2015 [45] Australia	Patient satisfaction and comfort levels of chronic disease services, based on survey data measuring patient satisfaction with nurse encounters and comfort with nurse roles in general practice	Patient satisfaction with PN services was very high, with nearly two-thirds (*n* = 51; 63%) of consumers giving the maximum score. However, no statistically significant group differences were detected between patient characteristics, number of visits to the nurse and satisfaction ratings Patient self-reported comfort was also high (median: 72, range: 18–90). Patients who consulted PNs for diabetes-related conditions were almost three times more comfortable (38% v. 14%, *p* = 0.016) with their encounter than those who consulted for other chronic health conditions
Marshall et al., 2011 [51] New Zealand	Patient satisfaction with health and treatment as measured by a consultation satisfaction survey	Of the 424 patients who completed a survey, 91% indicated they agreed or strongly agreed with the questions that stated a positive aspect of their care. Questions 3–5 specifically asked if health had improved as a result of attending clinics; 92% indicated that they agreed or strongly agreed. Ninety-four percent of patients had a better understanding of their diagnosis, medication and treatment plan, and were more motivated to self-manage
Zwar et al., 2010 [56] Australia	Patient feedback on their satisfaction with the quality of smoking cessation support they received during a 6-month follow-up questionnaire	Of the 391 participants who responded to the patient satisfaction questionnaire, 385 (98%) rated the support provided as ‘helpful’ (19%) or ‘very helpful’ (79%). Less than 2% commented that the program could have been improved and all comments indicated that they may have been more successful if they had been able to have more sessions with the RN
**Patient-Reported Outcome Measures (PROMs)**		
Aubert et al., 1998 [43]	Episodes of severe hypoglycemia; emergency room and hospital admissions	There were no statistically significant differences between nurse case management groups and usual care for adverse events
	Patient health-related quality of life as assessed by a questionnaire developed across four domains: 1) patient-perceived general health status, 2) patient-perceived physical dysfunction during the previous 30 days, 3) patient-perceived mental dysfunction during the pervious 30 days, and 4) patient-perceived functional incapacity during the previous 30 days for either mental or physical reasons	Both groups reported an improved perception of health status after 12 months, but patients in the nurse case management group were more than twice as likely to report improvement in health status score (mean change = 0.47) than the usual care group (mean change = 0.20) (difference=0.27; 95% CI: -0.03 to 0.57;*p *= 0.02)
Cherkin et al., 1996 [57] USA	Physical and social function as measured by a modified version of the Roland Disability Questionnaire, including questions that pertained to back and leg pain Disability as measured by an adaptation to the National Health and Interview Survey, which was implemented at 1, 3 and 7 weeks	There was no statistically significant increase in function or decreases in disability. The proportion of subjects reporting any days of limited activity, bed rest, or work loss resulting from their back pain was similar in all groups at each follow-up interval
Harris et al., 2015 [59] UK	Changes to patient self-reported levels of depression, anxiety, and pain as measured by questionnaire responses at 3 and 12 months	There were no statistically significant between-group differences in mean scores of depression, anxiety, or pain at 3 or 12 months
	Falls, fractures, sprains, injuries, or any deterioration of health problems already present at 3 and 12 months	There were no between-group differences in number of adverse events at 3 or 12 months
Harris et al., 2017 [60] UK	Changes in patient self-report outcomes of anxiety, depression and pain at 3 and 12 months	The interventions had no significant effects on anxiety, depression, or pain scores
	Falls, injuries, fractures, cardiovascular events, and deaths at 3 and 12 months	Total adverse events did not differ between groups at 3 or 12 months, however, cardiovascular events over 12 months were lower in the intervention groups than in controls (*p* = 0.04)
	Changes in patient-reported outcomes of exercise self-efficacy and quality of life at 3 and 12 months	Exercise self-efficacy significantly increased in both intervention groups at 3 months for postal group v. control group (ES = 1.1, 95% CI: 0.2 to 2.0; *p* = 0.01), nurse group versus control (ES = 2.3, 95% CI: 1.4 to 3.2; *p* < 0.001) and there was a greater effect in the nurse group compared with postal (ES = 1.2, 95% CI: 0.3 to 2.1;* p* = 0.01). By 12 months, there was a difference between only the nurse and control groups (ES = 1.2, 95% CI: 0.3 to 2.2, *p* = 0.01). The interventions had no significant effects on quality of life scores
Iles et al., 2014 [46] Australia	Patient quality of life measured by patient questionnaires at baseline (pre-intervention) and at 2 years, including a quality of life score using the EuroQol 5-Dimensions, scored with the Australian algorithm	Patient quality of life scores did not differ at baseline between RN-led groups (0.81 ± 0.18) and GP-led groups (0.81 ± 0.18). The quality of life score was inversely associated with MBS item charges (*p* < 0.001). On average, a 1% increase in the quality of life score resulted in a 44.5% decrease in MBS item charges
Marshall et al., 2011 [51] New Zealand	Patient physical fitness, daily activity, social activity, social support, feelings, and quality of life, derived from the Dartmouth Primary Care and Cooperative charts and patient self-report survey data	Significant improvements were shown in survey results for social activity (mean difference = -0.20; *p *= 0.049), change in health (mean difference = -0.42; *p* = 0.001), and overall health (mean difference = -0.21; *p* = 0.025); there no changes were reported for quality of life
Moher et al., 2001 [52] UK	Patient self-report quality of life, as measured by the Dartmouth Primary care and Cooperative charts and the EuroQol questionnaire	The study found no significant or clinically important difference between groups for any dimension of the Dartmouth Primary Care and Cooperative charts or for EuroQol scores
Pearson et al., 2003 [61] USA	Changes in patient level of depression, overall physical and mental health, and the impact of depression on their work and productivity from baseline to 6-month follow-up	Significant differences between baseline and six months were seen in the major subscales of the Work Limitations Questionnaire: time demands: 66.5 to 84.2, physical demands: 84.1 to 91.3, mental demands: 63.7 to 83.6, interpersonal demands: 77.2 to 90.5, and work output: 67.7 to 85.3. Paired t-test results for the difference in mean scores at baseline and 6-month follow-up for the SF-12 (mean = 29.9 to 48.2), Hamilton Depression Rating Scale (mean = 14.6 to 6.5) and Work Limitations Questionnaire (mean = 70.4 to 87.2) were statistically significant at the 0.0001 level These results show a significant reduction in depression severity for patients treated by the nurse telecare program, with 63% experiencing at least 50% reduction in their score at the 6-month follow-up
**Health Behaviours**		
Aveyard et al., 2007 [53] New Zealand	Confirmed sustained abstinence from smoking at 4, 12, 26, and 52 weeks after quit day	Of the participants in the basic and weekly arms, the quit % and the percentage difference was 22.4% v. 22.4% at 4 weeks (OR = 1.00; 95% CI: 0.74 to 1.37), 14.1% v. 11.4% at 12 weeks (OR = 0.79; 95% CI: 0.54 to 1.17), 10.7% v. 8.8% at 26 weeks (OR = 0.81; 95% CI: 0.52 to 1.25), and 7.7% v. 6.6% at 52 weeks (OR = 0.85; 95% CI: 0.51 to 1.41). There was no evidence that those in the weekly contact arm were more likely to quit, with point estimate of the quit rates favoring the basic support arm Absolute quit rates achieved are those expected from nicotine replacement therapy alone; neither of the support types were considered effective. PNs have a key role in providing support for smoking cessation, however, providing basic medication support is an adequate approach to achieve positive outcomes
	Patient reported use of nicotine replacement therapies at first telephone call and at each follow-up contact	Rates of nicotine replacement therapy use were high and did not differ between arms
Byers et al., 2018 [54] USA	Smoking status changes (i.e., whether patients reported themselves as smokers or non-smokers [former, quit, etc.]) at their last visit compared to their first visit	In GP-led visits, 18.2% (14 out of 77) patients who were reported as smokers during their first visit were reported as nonsmokers at their last visit, compared with 29.1% (41 out of 141) patients who attended RN-led visits. This difference was not statistically significant (*p* = 0.077); however, the findings suggest that smoking cessation is at least equivalent in patients who attend nurse-led visits compared with physician-led visits, and may be higher
Cherkin et al., 1996 [57] USA	Changes to patient self-reported participation in regular aerobic exercise between baseline and follow-up	Self-reported exercise was higher in the nurse intervention group after a 1-week follow-up (*p* < 0.001), however, there was no significant difference after 7 weeks
Faulkner et al., 2016 [55] UK	Self-reported 2-week point prevalence smoking abstinence at the 8-week follow-up Self-reported 6-month prolonged smoking abstinence at 6 months follow-up CO2 verified 2-week point-prevalence smoking abstinence at 4 weeks following quit date	No statistically significant differences between the two groups in the primary outcome measure of 2-week point prevalence abstinence at 8 weeks follow-up in both the unadjusted (OR = 1.01, 95% CI: 0.73 to 1.40) and adjusted models (OR = 1.07, 95% CI: 0.76 to 1.51) (adjusted for patients’ occupational category, initial CO reading and trial intervention arm) There were also no statistically significant differences in abstinence for support delivered by HCAs v. nurses at 4 weeks (unadjusted OR = 1.15, 95% CI: 0.80 to 1.66; adjusted OR = 0.86, 95% CI: 0.52–1.40) or 6 months follow-up (unadjusted OR = 0.86, 95% CI: 0.52 to 1.40; adjusted OR = 0.93; 95% CI: 0.55 to 1.56). Nurses and HCAs appear to be equally effective at supporting smoking cessation, however, nurses appear to be able to provide equivalent care with less patient contact
Harris et al., 2015 [59] UK	Daily physical activity as defined by change in average daily step-counts between baseline and 3 months, and between baseline and 12 months, assessed by accelerometry	At 3 months, changes in average daily step-counts were significantly higher in the intervention than control group by 1,037 (95% CI: 513 to 1,560; *p* < 0.001) steps/day. At 12 months, corresponding differences were 609 (95% CI: 104 to 1,115; *p* = 0.018) steps/day
	Weekly physical activity as defined by change in average weekly time spent in MVPA; MVPA in > 10-min bouts; accelerometer counts and counts per minute of wear-time between baseline and 3 months	The intervention increased objectively measured physical activity levels in older people at 3 months, with a sustained effect at 12 months. At 3 months, changes in weekly MVPA in ≥ 10-min bouts were significantly higher in the intervention than control group by 63 (95% CI: 40 to 87; *p* < 0.001) minutes/week, respectively. At 12 months corresponding differences were 40 (95% CI: 17 to 63; *p* = 0.001) minutes/week. Counts and counts/minute showed similar effects to steps and MVPA
Harris et al., 2017 [60] UK	Changes to physical activity as defined by average daily step counts, changes in step counts between baseline and 3 months, changes in time spent weekly in MVPA in > 10-min bouts, and time spent sedentary between baseline, 3 months and 12 months	Both intervention groups increased their step counts at 12 months compared with control (*p* < 0.001), with no statistically significant difference between nurse and postal delivery groups There were significant differences for change in step counts at the 3-month follow-up between intervention groups and the control group (nurse-supported group v. control 1,172 steps, 95% CI: 844 to 1,501; *p* < 0.001; postal group v. control 692 steps, 95% CI: 363 to 1,020;* p* < 0.001), however, the difference between the intervention groups was statistically significant (481 steps 95% CI: 153 to 809; *p* = 0.004). The two intervention groups had significantly increased step counts at 12 months, as compared to the control, but the two intervention groups did not significantly differ from each other on this outcome at 12 months. Findings for MVPA showed a similar pattern. The intervention had no significant impact on sedentary time
	Changes in patient-reported outcomes of exercise self-efficacy and quality of life at 3 and 12 months	Exercise self-efficacy significantly increased in both intervention groups at 3 months for postal group v. control group (ES = 1.1, 95% CI: 0.2 to 2.0; *p* = 0.01), nurse group versus control (ES = 2.3, 95% CI: 1.4 to 3.2; *p* < 0.001) and there was a greater effect in the nurse group compared with postal (ES = 1.2, 95% CI: 0.3 to 2.1; *p* = 0.01). By 12 months, there was a difference between only the nurse and control groups (ES = 1.2, 95% CI: 0.3 to 2.2, *p* = 0.01). The interventions had no significant effects on quality of life scores
Marshall et al., 2011 [51] New Zealand	Changes in smoking status (including both smoking cessation as well as smoking reduction) between first and last clinic attended	Although the percentage of adults who reported smoking remained the same between the first and last clinic data, there was a change in number of cigarettes smoked, in that the percentage of people who smoked between 0 and 10/day increased and those who smoked ≥ 11/day decreased
	Patient physical fitness, daily activity, social activity, social support, feelings, and quality of life, derived from the Dartmouth Primary Care Cooperative Information charts and patient self-report survey	Significant improvements were shown in survey results for social activity (*p* = 0.049), change in health (*p* = 0.001), and overall health (*p* = 0.025); there no changes were reported for quality of life. Ninety-four percent of patients reported having a better understanding of their diagnosis, medication and treatment plan, and that they were more motivated to self-manage their health needs.
Pine et al., 1997 [49] USA	Changes to patient dietary intake as measured by the EPAT from first to final nurse visit	Mean EPAT scores at baseline in both studies demonstrated that intervention patients were already following a diet consistent with the National Cholesterol Education Program Step 1 Diet. However, the mean Section 1 EPAT score improved from 23.4 at the first nurse visit to 20.4 at the final nurse visit (*p*< 0.001)
Zwar et al., 2010 [56] Australia	Smoking status, defined as “point prevalence” (no smoking in seven days preceding the assessment) and “continuous abstinence” (no smoking from quit date to assessment at 4- and 6-month follow-up)	At 6-month follow-up, the point-prevalence abstinence rate was 21.7% (108 out of 498 participants at baseline) and the continuous abstinence rate was 15.9% (79 out of 498 participants at baseline). Participants with very low to medium nicotine dependence (0–5 Fagerström Score) had significantly higher point prevalence cessation rates than those with high to very high dependence (score > 5) (*p* < 0.001). Continuous abstinence rate was not significantly different between these groups. Patients who had attended four or more counseling visits with the RN were significantly more likely to quit at 6 months than patients who attended less than four times (point prevalence abstinence 32% v. 9%, *p* < 0.001; continuous abstinence 25% v. 3%, *p* <0.001)

*HbA1c* hemoglobin A1c, *RN *registered nurse, *BMI *body mass index, *CHD *coronary heart disease, *PN *practice nurse, *OR* odds ratio, *CI* confidence interval, *CPS *clinical pharmacy specialist, *BP *blood pressure, *ES* effect size, *cmH*_2_0 centimetres of water pressure, *GP *general practitioner, *HCA *health care assistant, *MBS *Medicare Benefits Schedule, *MVPA *moderate to vigorous physical activity, *EPAT* Eating Pattern Assessment Tool

### Physiologic disease control via biomarkers

Ten studies measured clinical patient outcomes when comparing interventions that involved primary care RNs to that of usual care or an intervention delivered by a comparator group. Clinical biomarkers included those for diabetes (HbA1c, fasting blood glucose), obesity (BMI, total fat mass), hypertension (blood pressure), and cardiovascular risk (total cholesterol). Of the ten studies, four examined diabetic control. After one year, Aubert et al [43]. reported significant differences in HbA1c values; patients in the primary care RN case management group had a larger mean reduction (-1.7 percentage points) over 12 months in comparison to usual care (-0.6 percentage points) (difference -1.1, 95% CI: -1.62 to 0.58; *p* < 0.001). Additionally, patients in the intervention group had a greater decrease in fasting blood glucose than the usual care group (-48.3 mg/dL versus -14.5 mg/dL; difference -33.8, 95% CI: -56.12 to 11.48; *p* = 0.003). Bellary et al [44]. found a small but non-significant reduction in HbA1c among their patient sample after two years. One additional study that conducted a retrospective data analysis of clinical outcome data from patients attending an independently RN-led primary care clinic, did not detect significant changes in HbA1c between initial intake at baseline and follow-up visits at various intervals (reported as 3 months to “several years” depending on the individual) [51].

Seven studies examined obesity-related outcomes such as BMI, weight, and total fat mass. In their adjusted regression models, Karnon et al [47]. reported that high level involvement of primary care RNs in the provision of obesity-related clinical activities (in comparison to low level involvement) yielded significantly larger mean reductions in BMI (mean difference -1.10, 95% CI: -0.45 to -1.76; *p *= 0.001) after one year, however, there were no significant improvements in terms of the proportion of patients losing weight (mean difference 0.09, 95% CI: -0.07 to 0.25; *p* = 0.259). Coppell et al [50]. found a significant weight reduction (-1.3 kg) in the primary care RN-led prediabetes intervention arm compared to usual care (gained 0.8 kg) (2.2 kg difference; *p* < 0.001). Mean BMI and waist circumference also decreased in the intervention arm compared to an increase in the control group, however, these differences were not significant. Likewise, a third study reported that fat mass was slightly reduced at 12 months, but differences between the intervention and control groups were equivalent when the primary care RN group was compared to both postal intervention (*p* = 0.54) and usual care (*p* = 0.30) [60]. There were no significant reductions in BMI or waist circumference in the remaining four studies [43, 44, 51, 59].

Five studies investigated the impact of enhanced nurse involvement in primary care delivery on blood pressure. Bellary et al [44]. reported significant differences between groups in diastolic blood pressure (-1.91, 95% CI: -2.88 to -0.94 mm Hg; *p *= 0.0001) and mean arterial pressure (1.36, 95% CI: -2.49 to -0.23 mm Hg; *p* = 0.018), favoring the intervention (additional time spent with a primary care RN). In a second study conducted by O’Neill et al. in which the RN independently assessed blood pressure and collaborated with either a CPS or physician in hypertension case management, [48] there was a greater decrease in systolic blood pressure in patients who received care from CPS and primary care RN teams (-14 mm Hg) compared to patients receiving care from physician-directed primary care RNs (-10 mm Hg) (*p* = 0.04), however, there were no significant changes in diastolic blood pressure between groups. The remaining three studies found no significant changes in blood pressure when comparing a primary care RN-led intervention to that of usual care [43, 50] or from initial baseline to follow-up [51].

Total cholesterol was measured in four studies. Pine et al  [49]. reported that the mean total cholesterol level decreased by 0.29 mmol/L (11.2 mg/dL) (4.3%) from the initial physician visit to the first primary care RN visit. Following five counseling sessions by a primary care RN, the mean total cholesterol levels of all patients decreased (-0.14 mmol/L; *p* = 0.4). However, during the follow-up comparison study, there were no significant differences in total cholesterol improvement between the nurse-counseling intervention group and the comparison patients, and total cholesterol levels in both groups improved significantly (*p* = 0.002). The remaining three studies reported equivalent results in regards to total cholesterol reduction [43, 44, 50].

### Patient experience outcomes via PREMS

Nine articles reported on patient experience outcomes via PREMs: overall perceived quality of care [45, 51, 55, 63, 65], self-management support [56, 62], access (first point of primary care contact) [64], comprehensiveness [57], and trust [45].

In regard to overall perceived quality of care, Halcomb and colleagues [45] found that Australian patients were very satisfied and comfortable with chronic disease care delivered by a primary care RN. This was particularly true for patients with diabetes who reported being almost three times more comfortable (38% versus 14%, *p* = 0.016) with their encounter than patients who consulted for other chronic health conditions. A similar study in New Zealand also revealed high satisfaction with primary care RN-delivered services overall, with increased satisfaction associated with an increased number of visits (i.e., those who had more than four previous visits to the primary care RN) after controlling for demographic factors [63]. Longer consultation time with a primary care RN resulted in higher patient satisfaction (OR = 2.50, 95% CI: 1.43 to 4.35;* p* < 0.01) and patient enablement (OR = 2.55, 95% CI: 1.45 to 4.50; *p* < 0.01) than shorter consultation time [62]. Moreover, patients who attended practices where primary care RNs worked with broad scopes of practice and high levels of autonomy were more satisfied (OR = 1.76, 95% CI: 1.09 to 2.82; *p* = 0.04) and more enabled (OR = 2.56, 95% CI: 1.40 to 4.68; *p* < 0.01) than patients who attended practices where nurses worked with more limited scopes of practice and lower levels of autonomy [62].

Patients also reported improved health, better understanding of disease diagnosis, medication, and treatment plan, and more motivation for self-management as a result of primary care RN-led lifestyle clinics focused on diabetes, smoking cessation, women’s health, cardiovascular risk, respiratory/asthma, and diet/nutrition [51]. Furthermore, patients reported positive experiences with primary care RN-led telephone consultations for acute illness [65], back pain education [57], and smoking cessation support [55, 56]. For instance, Cherkin et al [57]. reported higher satisfaction (*p *< 0.0) and higher perceived knowledge (*p* < 0.001) for patients who received a primary care RN-led educational intervention for back pain than those in the usual care group. Nearly all patients (98%, *n* = 385) in an Australian study [56] that examined smoking cessation behavioral support from a primary care RN rated the support provided as helpful (19%) or very helpful (79%) and indicated that they may have been more successful with smoking cessation if they had been able to have more sessions with the RN. With respect to access to care, a study by Caldow et al [64]. found that patients expressed satisfaction and preference with primary care RN versus physician consultations for minor illness as first point of contact if this resulted in a reduced waiting time, suggesting that patients would be accepting of an expanded nursing role in primary care.

### Patient reported outcomes via PROMs

Patient reported outcome measures via PROMs were examined across eight studies and included health-related quality of life [43, 46, 51, 52, 60], symptoms [59–61], self-efficacy [60], and functional status [57].

Health-related quality of life, as measured through patient self-report, was assessed in five studies [43, 46, 51, 52, 60]. In a 12-month randomized controlled trial conducted by Aubert et al., [43] a primary care RN-led case management model of adult diabetes care was compared with that of usual care in a primary care setting. Health-related quality of life was assessed by a validated questionnaire developed by the Centers for Disease Control and Prevention for the Behavioral Risk Factor Surveillance System to assess patient perception of health status across four domains. The results demonstrated an improved perception of health status in both groups, with patients in the intervention group more than twice as likely to report improvement in health status score (mean change = 0.47) as those in the usual care group (mean change = 0.20) ( difference= 0.27; 95% CI: -0.03 to 0.57; *p* = 0.02). In contrast, the other four studies [46, 51, 52, 60] examining health-related quality of life did not report significant differences in regards to these outcomes, including two cluster randomized controlled trials [46, 52, 60]. One study assessed the effectiveness of three different methods of secondary prevention care of coronary heart disease (recall to a primary care RN; recall to a physician; audit and feedback) [52], while the other compared the efficacy of a primary care RN-supported physical activity intervention to that of usual care [60]. Both studies reported equivalent scores between groups on all dimensions of patient self-reported quality of life measurements. Lastly, an observational study by Marshall et al [51]. used patient satisfaction surveys to assess perceptions of Nurse-Led Healthy Lifestyle Clinics (NLHLC) in New Zealand. Using scores from the Dartmouth Primary Care Cooperative (COOP) Information charts [66], it was noted that there were no statistically significant differences in the COOP dimensions related to self-perceived quality of life from first clinic visit to last clinic visit. However, significant improvements were noted in relation to the COOP variables related to patient-perceived social activity (mean difference = -0.20; *p* = 0.049), change in health (mean difference = -0.42; *p* = 0.001), and overall health (mean difference = -0.21; *p* = 0.025).

Patient self-reported symptoms were measured in three studies [59–61] and included outcomes related to both mental and physical health (anxiety, depression, pain), as well as the occurrence of adverse health events (injuries, fractures, cardiovascular events, deaths, and deterioration of any pre-existing health problems). In an observational study of a nurse telecare intervention for adults with depression in the United States, Pearson et al [61]. found a significant improvement in mean scores on the SF-12 Mental Functioning Scale between baseline (mean = 29.9) and 6-months post-intervention (mean = 48.2) (*p* < 0.0001). During the same time interval, significant differences were noted on the Hamilton Depression Rating scale (14.6 to 6.5; *p* < 0.001), as well as the mean scores on the Work Limitations Questionnaire (70.4 to 87.2; *p* < 0.001), which both represent an improvement in functioning. Paired t-test results for the difference in mean scores on all three instruments were statistically significant (*p *= 0.0001) and the majority of patients (63%) experienced at least a 50% reduction in the Hamilton Depression Rating score at 6-months. The remaining studies to examine self-reported symptoms as an outcome were two randomized controlled studies that measured the effects of a primary care RN-delivered intervention on patient physical activity [59, 60]. Both studies assessed changes to patient self-reported levels of depression, anxiety, and pain and incidents of adverse health events. The results of both studies reported no statistically significant between-group differences in mean scores of either symptom at 3- or 12-months post-intervention. Additionally, while total number of adverse events did not differ between groups for either study, Harris et al [60]. found a significant reduction in cardiovascular events among the intervention group over the 12 month period (*p* = 0.04).

Patient self-efficacy was examined in a three-arm cluster randomized controlled study conducted by Harris et al [60]., in which patient-reported levels of exercise self-efficacy were examined at 3- and 12-months following a physical activity intervention. Exercise self-efficacy in this study was characterized by a patient’s willingness to set goals, create action plans, engage in self-monitoring, and seek out social support, and are directly related to long-term physical activity adherence. Findings indicated that exercise self-efficacy was significantly increased in both intervention groups at 3-months for postal group (pedometer delivered by mail) versus control (Effect Size [ES] = 1.1, 95% CI: 0.2 to 2.0; *p* = 0.01), primary care RN group versus control (ES = 2.3, 95% CI: 1.4 to 3.2;* p *< 0.001), and primary care RN group versus postal group (ES = 1.2, 95% CI: 0.3 to 2.1; *p* = 0.01). For primary care RN group versus control group, the difference remained significant at the 12-month follow-up (ES = 1.2, 95% CI: 0.3 to 2.2; *p* = 0.01), but not for the postal group versus control (*p* = 0.2) or the primary care RN group versus postal group (*p* = 0.22).

The sole study to evaluate functional status was a randomized controlled trial conducted by Cherkin et al [57]. comparing usual care, usual care plus an educational booklet, and usual care plus an educational session with a primary care RN and an educational booklet to improve outcomes of low back pain in primary care. None of the interventions had a statistically significant effect on functional status, including days of limited activity, bed rest, or work loss resulting from back pain one week after the intervention or at any subsequent follow-up.

### Health behaviors

Of all the studies included (*n* = 23), nine considered health behavior outcomes. Among the studies examining the impact of a primary care RN-led intervention on health behaviors, it was found that tobacco use was the most documented health behavior (*n* = 5) [51, 53–56]. Tobacco use was examined by looking at both abstinence from smoking, as well as daily reductions or changes to smoking behaviour, and this was measured at multiple follow-up periods throughout the duration of the intervention. All five studies demonstrated positive changes in smoking-related health behaviors following either an independent [51, 53–55] or interdependent [56] primary care RN intervention. For example, Byers et al [54]. compared a primary care RN-led intervention with a physician-led intervention to support smoking cessation. The results show that support provided by the primary care RN was equivalent to that provided by the comparator group (29.1% versus 18.2% quit rate, respectively; *p* = 0.077) in supporting the patient to quit smoking. Marshall et al [51]. looked at primary care RN-led healthy habits lifestyle clinics for patients with or at risk of chronic disease within targeted populations with known health inequalities. Following the intervention, 94% of patients reported having a better understanding of their diagnosis, medication and treatement plan, and an increase in motivation to self-manage their health needs. Other studies have examined the impact of nursing interventions on patient-reported levels of physical activity [57, 59, 60]. Only one study considered adherence to healthy eating as a health outcome following a RN-led intervention in primary care [49]. In this study, Pine and colleagues evaluated the effect of a nursing intervention to support cholesterol lowering for patients diagnosed with hypercholesterolemia. To do this, primary care RNs provided a total of five counseling visits focused on nutritional education and physical activity (1-month after referral, and at 3-, 5-, 7-, and 12-months) to 82 patients with total cholesterol higher than 6.21 mmol/l. Intervention patients were already following a diet consistent with the program at baseline, however, the mean score for Section 1 of the Eating Pattern Assessment Tool (questions related to foods with serum cholesterol-raising potential) improved from 23.4 at the first visit to 20.4 at the final visit (*p* < 0.001).

## Discussion

This systematic review presents a comprehensive synthesis of literature examining the impact of primary care RNs on patient outcomes. The findings suggest that outcomes resulting from care provided by primary care RNs are comparable and complementary to care provided by other primary care providers, specifically with respect to chronic disease prevention and management, smoking cessation, and wellness counseling. This review supports that primary care RNs deliver appropriate and high-quality patient care. There was a high level of patient satisfaction reported regarding experiences with RN-led care. Patients appear to be comfortable with RNs providing primary care services and taking on expanded roles in primary care. This is consistent with findings from other studies that have examined patient satisfaction and comfort with RN roles in primary care practices across multiple countries [67–70]. Our findings are aligned with existing evidence that has linked patient experiences of care to the level of autonomy and scope of practice of the RN in the clinical setting. A recently published study from Canada evaluated patient experiences in primary care organizations and determined that patient-reported experience was significantly enhanced in clinics in which RNs systematically followed patients and used their scope of practice to their full potential [71].

Patient experience is a strong indicator of patient-perceived quality of care and fundamental to achieving desired patient outcomes for a range of physical and mental health domains [72–76]. The recently developed PaRIS Framework served as a valuable tool for organizing patient-reported outcomes, however, it did not capture all patient outcomes identified within the studies included in this review. For example, clinical biomarkers, such as HbA1c and fasting blood glucose (used as a measure of diabetes care quality), were not considered in the PaRIS Framework and therefore added by the study authors during the analysis phase as an additional patient outcome category. Similarly, studies evaluating RN interventions in primary care did not consider many components of the PaRIS Framework, such as delivery system design (e.g., clinic remuneration, remote consultations), individual and sociodemographic factors (e.g., patient demographic characteristics), and health and health care capabilities, and only measured select components from the patient-reported experiences of care, health behaviours, and patient-reported outcome domains. For instance, many articles did not provide information regarding RN characteristics, such as level of education, years of experience, or specific roles/tasks that they performed in-clinic prior to the intervention that might have impacted outcomes observed. In addition, conceptual definitions of outcomes within included studies may vary and not align with meanings as defined within the PaRIS Framework. A taxonomy for RN outcomes may be useful and should be considered in future revisions and applications of the OECD PaRIS Framework. 

The included studies evaluated a variety of primary care RN interventions but did not capture all roles that encompass their broad scope of practice. Commonly offered services by primary care RNs that have yet to be comprehensively evaluated include prenatal and well-baby care, therapeutic interventions (e.g., wound care, treatment of infections), preventative care (e.g., immunizations, health promotion and education) and care coordination (e.g., nursing surveillance, system navigation). Although self-management supports (e.g., smoking cessation, physical activity, diabetes, nutrition, pain management, healthy lifestyle promotion, chronic disease prevention) were examined in a few studies [43, 44, 49–51, 53–57, 59, 60], further evaluation of self-management and behaviour support interventions (included within the PaRIS Framework) offered by RNs in primary care is needed. Moreover, recently developed competencies identify that, in addition to clinical practice activities centered around patient care provision, primary care RNs engage in a wide range of non-clinical roles, such as leadership, research, and interprofessional collaboration. These non-clinical domains of practice for primary care RNs require further understanding and evaluation.

There is a general apprehension among some medical practitioners that if RNs assume more responsibilities or enhanced roles within primary care settings, high-quality care and patient safety will be compromised [77, 78]. Findings from this study show that patient care is equivalent to that of “usual care” and in many cases, produced better patient outcomes when the intervention was provided by a primary care RN. This aligns with literature in the acute care and long-term care settings [24, 25]. The findings from our review call into question concerns that RN-provided care increases risk or reduces quality of care and equally, lends support towards the efficacy of primary care RN care provision on improvements to patient outcomes. Additionally, primary care RNs are required to practice within their legislated and regulated scope of practice, regardless of the practice setting or types of clinical roles performed [79, 80].

Generally, there are methodological challenges associated with examining the contributions of a specific health care provider within the context of a team [81–83]. As the focus of practice and research moves towards interdisciplinary teams, it is increasingly difficult to isolate and evaluate the impact of primary care RN interventions. In addition, the roles of primary care RNs and team compositions vary across practice settings. Although this review exclusively examined studies in which an intervention was delivered by a RN, many studies were excluded because of unclear terminology surrounding the nursing designation/role (i.e., unable to discern whether nurse in study was a RN or equivalent). Furthermore, the review included only nine randomized controlled trials, which provide the strongest level of evidence, that specifically compared RN-led interventions to care delivered by other health care professionals and/or usual care [43, 52, 53, 57–60, 84, 85]. Comparator groups in studies varied considerably, impacting the ability to make comparisons across studies and limiting the generalizability of findings from this study. Despite these challenges, this review provides preliminary evidence on patient outcomes used to evaluate a variety of different RN interventions in a multidimensional health care environment. The findings from this study, coupled with an existing framework (e.g., OECD PaRIS Framework) serve as a tool to map roles and activities to outcomes and guide future evaluation of primary care RNs. Overall, as the presence of RNs in primary care increases globally, further evaluation research implementing control/comparison groups into study design and controlling for confounding factors (e.g., nurse characteristics) is needed to strengthen the evidence related to the effectiveness of RNs in primary care.

### Strengths and limitations

This review provides preliminary evidence regarding the effectiveness of RNs on patient outcomes in primary care. Traditional means of measuring the effectiveness of care provision in the healthcare sector have focused mainly on the use of clinical data. A strength of this systematic review is its patient-oriented approach that assesses health outcomes from the patient perspective [27, 86]. Additional strengths of this systematic review include the application of a comprehensive search strategy and use of the PRISMA checklist in the screening process. However, despite utilizing a comprehensive search strategy, it is possible that not all relevant studies were retrieved and included in this review. Furthermore, although we conducted an appraisal using an established quality assessment tool (i.e., ICROMS), this tool presented certain challenges. For instance, although a strength of this tool is that it offered criteria to assist with the process of assigning quality scores, there is a degree of subjectivity involved in the appraisal process. In addition, the minimum cut-off scores varied across study designs and therefore, made comparisons of the quality between different types of studies difficult. Similarly, the score itself is difficult to interpret without an understanding of the tool and design matrix (limitations of articles are summarized in Supplementary Table 3). The ICROMS tool was also not designed to specifically appraise mixed methods or observational designs. The lack of consistent and available data regarding terminology used to describe RNs, or equivalent nursing titles, across countries limited the ability to include studies published in certain regions. Only studies published in the English language were included, which may limit generalizability to certain countries. High-quality research employing robust study designs (e.g., randomized controlled trials) need to be conducted to further understand the impact of RNs on patient outcomes in primary care.

## Conclusions

Primary care RNs can provide patient care that is comparable and complementary to that of other primary care providers, specifically with respect to patient satisfaction, enablement, self-reported quality of life, self-efficacy, and improvements in health behaviours. This review provides preliminary evidence regarding the effectiveness of RNs on patient outcomes in primary care. Findings are applicable to researchers and other stakeholders engaged in primary care reform and can inform further integration and optimization of this role, as well as contribute to future research.

## Supplementary Information

**Additional file 1.**

**Additional file 2.**

**Additional file 3.**

## Data Availability

All data generated or analysed during this study are included in this published article.

## Abbreviations

RNs: Registered Nurses
PaRIS: Patient Reported Indicator Survey
PREMs: Patient-reported experience measures
PROMs: Patient-reported outcome measures
JBI: Joanna Briggs Institute
PRISMA: Preferred Reporting Items for Systematic Reviews and Meta-Analysis
PROSPERO: Prospective Register of Systematic Reviews
ICROMS: Integrated Quality Criteria for Review of Multiple Study Designs
HbA1c: Hemoglobin A1c
BMI: Body mass index
CPS: Clinical Pharmacy Specialist
COOP: Primary Care Cooperative
NLHLC: Nurse-Led Healthy Lifestyle Clinic
OECD: Organization for Economic Cooperation and Development
ES: Effect Size
