# Correction: Effectiveness of registered nurses on patient outcomes in primary care: a systematic review

**DOI:** 10.1186/s12913-022-08204-x

**Published:** 2022-06-22

**Authors:** Julia Lukewich, Ruth Martin-Misener, Allison A. Norful, Marie-Eve Poitras, Denise Bryant-Lukosius, Shabnam Asghari, Emily Gard Marshall, Maria Mathews, Michelle Swab, Dana Ryan, Joan Tranmer

**Affiliations:** 1 Faculty of Nursing, Memorial University, 300 Prince Phillip Drive, St. John’s, NL A1B 3V Canada; 2 School of Nursing, Dalhousie University, 5869 University Ave. St, Halifax, NS B3H 4R2 Canada; 3 School of Nursing, Columbia University, 630 West 168th Street, New York, NY 10032 USA; 4 Département de Médecine de Famille Et Médecine d’urgence, Université de Sherbrooke, 2500 Boulevard de l’Université, Sherbrooke, QC J1K 2R1 Canada; 5 School of Nursing, McMaster University, 1280 Main St W, Hamilton, ON L8S 4L8 Canada; 6 Department of Family Medicine, Memorial University, 300 Prince Phillip Drive, St. John’s, NL A1B 3V6 Canada; 7 Department of Family Medicine Primary Care Research Unit, Dalhousie University, 1465 Brenton Street, Suite 402, Halifax, NS B3J 3T4 Canada; 8 Department of Family Medicine, Schulich School of Medicine & Dentistry, University of Western, 1151 Richmond Street, Ontario, London, ON N6A 5C1 Canada; 9 Health Sciences Library, Faculty of Medicine, Memorial University, 300 Prince Phillip Drive, St. John’s, NL A1B 3V6 Canada; 10 School of Nursing, Queen’s University, 92 Barrie Street, Kingston, ON K7L 3N6 Canada

**Correction: BMC Health Serv Res 22, 740 (2022)**

**https://doi.org/10.1186/s12913-022-07866-x**

Following publication of the original article [1], the authors identified a typesetting error in the third paragraph of the ‘Patient experience outcomes via PREMS’ subsection under the ‘Results’.

The sentence originally read:

For instance, Cherkin et al. [57] reported higher satisfaction **(p < 0.0)** and higher perceived knowledge (p < 0.001) for patients who received a primary care RN-led educational intervention for back pain than those in the usual care group.

The sentence should read:

For instance, Cherkin et al. [57] reported higher satisfaction **(p < 0.01)** and higher perceived knowledge (p < 0.001) for patients who received a primary care RN-led educational intervention for back pain than those in the usual care group.

The original article [1] has been corrected. The publisher apologises to the authors and readers for the inconvenience caused by the error.
