# Supplementary material for: Effectiveness of registered nurses on patient outcomes in primary care: a systematic review
Source: BMC Health Serv Res. 2022 Jun 3;22:740. doi: 10.1186/s12913-022-07866-x (PMC9166606; doi:10.1186/s12913-022-07866-x)
Supplement: Supplementary file 2 — Additional file 2. [file 12913_2022_7866_MOESM2_ESM.docx]

**Supplementary File 2. Decision Matrix Outlining Mandatory Criteria and Minimum Score for Study Type to be Included in Review Based on ICROMS Quality Appraisal Tool***

| **Study Design^a^** | **Mandatory Criteria** | **Minimum Score^b^** |
| --- | --- | --- |
| RCT, cRCT | 1A, 2A, 2B, and 3A | 22 |
| CBA | 1A, 2D, 3B, and 3C | 18 |
| CITS | 1A, 3D, and 6A | 18 |
| NCITS | 1A, 1B, 2C, and 5D | 22 |
| NCBA | 1A, 1B, 2C, and 5D | 22 |
| CS | 1A, 2E, 3G, and 4C | 18 |
| Obsv | 1A, 2C, and 2F | 16 |
| **-Zingg W, Castro-Sanchez E, Secci FV, Edwards R, Drumright LN, Sevdalis N, et al. Innovative tools for quality assessment: integrated quality criteria for review of multiple study designs (ICROMS). Pub Health. 2016;133:19-37. doi:10.1016/j.puhe.2015.10.012*  *a- Study Designs: RCT- randomized controlled trial; cRCT- cluster randomized controlled trial; CBA- controlled before-after; CITS- controlled interrupted time series; NCITS- non-controlled interrupted time series; NCBA- non-controlled before-after; CS- cohort study; Qual- qualitative study; Obsv- observational study*  *b- Scores applicable to each criteria: 2 points= Yes, criterion met; 1 point= Unclear whether or not the criterion was met; 0 points= No, criterion was not met.* | | |
