# Supplementary material for: Effectiveness of registered nurses on patient outcomes in primary care: a systematic review
Source: BMC Health Serv Res. 2022 Jun 3;22:740. doi: 10.1186/s12913-022-07866-x (PMC9166606; doi:10.1186/s12913-022-07866-x)
Supplement: Supplementary file 3 — Additional file 3. [file 12913_2022_7866_MOESM3_ESM.docx]

**Supplementary File 3. List of Study Limitations (n=23)**

| **Author Name, Year** | **Limitations** |
| --- | --- |
| Aubert et al., 1998 | ICROMS Score: 25  Study Design: Randomized controlled trial  -Only one RN (certified in diabetes education) involved in study; difficult to determine if results would be generalizable  -Intervention group had fewer members of ethnic minority groups, more smokers, more insulin-treated patients, and a larger number of patients with Type 1 diabetes  -Fairly high attrition rate (34% did not go to scheduled appointments; an additional 38 patients did not provide 12-month follow-up data). The majority of patients lost to follow-up were younger (18-44 years) and non-white  -Study involved implementation of an algorithm developed by researchers, the participation of physician advocates, and the RN to work closely with an endocrinologist and physician. It is difficult to discern whether the same results would be achieved under more ‘real world’ situations |
| Aveyard et al., 2007 | ICROMS Score: 23  Study Design: Randomized controlled trial  -Study did not examine long-term smoking abstinence (no data on whether smoking status changed after the 8-week period)  -Fairly high attrition rate (31%)  -Some practices employed different or additional recruitment techniques, possibly causing patient samples to differ (e.g., motivation, etc.)  -Number of exclusions/ineligible patients who were initially recruited was not recorded. Additionally, a small number of participants were later determined to have been ineligible based on rate of smoking (n=13) or type of smoking apparatus used (n=9), but were retained.  -Data collected on NRT use was recording whether or not NRT was being used in general, and not in the degree of adherence. Also, data from those with no contacts were excluded; thus, rates of NRT use may have been less than what was recorded.  -Participants and nurses were not blind to study group allocation  -There was a great deal of variation between practices in terms of care delivery (weekly support) and patient adherence; results may not be generalizable  -Study may be subject to information bias; the assessment of smoking status during the first 4 weeks was not identical (with more assessments in the weekly support arm) |
| Bellary et al., 2008 | ICROMS Score: 24  Study Design: Cluster randomized controlled trial  -More patients in the control group than in the intervention group were ex-smokers; more patients in the intervention group were being treated with statins  -RNs had protected time to run a research diabetes clinic and the support of diabetes specialist community nurses; may not be generalizable to “real world” care provision  -Study sample contained patients who were at a high cardiovascular risk, target HbA1c values may have been too difficult to achieve in this particular group  -Despite study protocol stating that all patients should be prescribed statins, only two-thirds were actually receiving them, which may have influenced the results  -Intervention contained many complex components (including care by link workers and a community nurse specialized in diabetes), it is difficult to assess the relative contributions of each individual component, specifically, the extent to which the RN role had on outcomes  -For patients with missing data, the authors employed the ‘last observation carried forward’ method, which is a method with known weaknesses |
| Byers et al., 2018 | ICROMS Score: 20  Study Design: Observational; retrospective secondary analysis  -Secondary analysis; data were not extracted for the purposes of smoking cessation analyses initially and methodology was not tailored to the examination of this outcome.  -Study duration was not clearly defined; due to limited resources, efforts to implement annual wellness visits occurred gradually in the participating clinics  -Primary care RNs were only used in 4 of the 6 participating clinics  -One visit was conducted by an advanced practice RN, however, this was considered to be part of the physician-led group  -Smoking status was not reported in 28.3% of the sample (with less reported in the physician group than the RN-led group)  -Groups were not matched for type of visit (RN-led visits were limited primarily to Medicare wellness visits, whereas physician-led visits were typically annual physicals with patients), making it difficult to draw comparisons  -Smoking status was determined by recorded data for first and last visit, however, it did not capture changes to smoking status throughout the study period |
| Caldow et al., 2006 | ICROMS Score: 21  Study Design: Observational; survey and telephone interviews  -Study duration was not indicated  -General known limitations associated with postal questionnaires are applicable  -Response rate for the questionnaire was somewhat low (49%)  -The patient condition/illness was not taken into consideration; it’s likely that preferences for seeing a RN vs. physician could be related to the patient’s perceived seriousness of the condition  -Analysis used a simple linear additive model, however, it would have been useful to extend analysis to allow for non-linearities in preference for service attributes and interaction with respondent characteristics |
| Cherkin et al., 1996 | ICROMS Score: 24  Study Design: Randomized controlled trial  -Study is somewhat dated (conducted from 1992-1993); changes may have taken place since then regarding methods of patient-centered educational interventions  -RNs in study had at least 20 years of clinical experience; this may not be generalizable to RNs with less experience.  -Attrition rate was fairly high (Only 34% of initial sample had at least one week follow-up data).  -Rather than standard randomization, “pre-consent randomization” was chosen  -Patient satisfaction ratings were obtained only at baseline and at a 1-week follow-up evaluation  -All outcomes were assessed by self-report measures; patients in the RN follow-up group may have felt compelled to embellish their level of physical activity/adherence to recommendations  -Participant sample was fairly uniform demographically (predominantly white, highly educated, employed, generally physically active); may not be generalizable to a wider population |
| Coppell et al., 2017 | ICROMS Score: 26  Study Design: Pragmatic, non-randomized controlled before-after; convergent mixed methods design  -Primary care RNs had regular follow-up with dieticians and a liaison nurse; difficult to determine whether results would be generalizable to standard practices within primary care  -Timely patient follow-up was somewhat affected when appointments fell in and around certain time periods (e.g., holiday seasons)  -Uptake of the community education component of the intervention was low; only 53% of intervention patients attended sessions and only one-third completed the course  -Sample may be prone to selection bias; RNs may have been more likely or comfortable approaching patients with whom they were familiar or already had a positive relationship with |
| Desborough et al., 2016 | ICROMS Score: 22  Study Design: Observational; cross-sectional survey  -Intervention involved 47 RNs and 3 enrolled nurses; it was not possible to discern who patients had seen and no effort was made to distinguish between them  -Continuity of care (i.e., patients requesting specific nurse) was difficult to measure, as this is not a typical service offered by most practices and patients are not accustomed to this  -Cross-sectional component limited the capacity to assess potential causal relationships between general practice, consultation characteristics and quality outcomes  -Certain variables (e.g., appointment length) used only patient self-report rather than an objective measure, which could result in an incorrect estimation  -The categories used to classify patients’ health conditions did not distinguish between chronic or acute and episodic care  -There was no information available about non-responders; it is possible that these individuals were more satisfied or enabled than those who did respond.  -Surveys were not always distributed consecutively; poor compliance was noted by reception and nursing staff due to the busy nature of the practices |
| Faulkner et al., 2016 | ICROMS Score: 21  Study Design: Cohort study using longitudinal data from a previously conducted randomized controlled trial  -Participants were required to have a mobile phone and the knowledge/ability to send and receive text messages; this may disproportionately affect patients from certain demographics (e.g., lower socioeconomic statuses, older patients, etc.)  -Relatively low uptake among practices approached (31%); sample of participating practices was not fully representative of areas of deprivation across England  -While the 4-week smoking outcome was biochemically verified, the 8-week and 6-month outcomes were measured by self-report only  -Study did not report on whether questionnaire was a validated tool or how it was developed  -Study should have followed up with participants beyond 6 months and had their abstinence biochemically verified in order to confirm long-term smoking abstinence  -Data was collected from patients 8 weeks after the initial consultation, increasing the risk of recall bias or erroneous recall  -The type smoking cessation advisor seen by participants was not determined at random. While participants in each group had similar characteristics, there may have been unmeasured patient or advisor cofounders that impacted findings |
| Gallagher et al., 1998 | ICROMS Score: 16; 22*  Study Design: Observational (cross-sectional) and uncontrolled before-after  -Intervention was carried out by only one RN who had 15 years of clinical experience and was familiar with and trained in conducting telephone consultations; may not be representative  -Some data was excluded due to poor information capture  -Postal questionnaire assessing patient satisfaction was only sent to patients who had received telephone advice from an RN, limiting the ability to make comparisons  -Physician workload reduction could be attributed to the season (summer months, “holiday period”), and thus a decline in overall patients; analysis compared workload during this time with the three months prior, rather than during the same season of the previous year. The period of data collection was too short to reveal diurnal and seasonal variations in acute illness workload  -There was a delay of 10 months in conducting the postal survey, which may affect the validity of the questionnaire findings and present issues with recall |
| Halcomb, Davies, & Salamonson, 2015 | ICROMS Score: 21  Study Design: Observational; descriptive survey  -Study does not describe the degree of autonomy/scope of practice that RNs had at each clinic  -Data gathered in study was entirely based on self-report  -Having the RN ask patients to complete the survey while in the clinic and immediately after their appointment could bias the patient towards more favourable responses (i.e., social desirability)  -The study was not able to determine a precise participation rate due difficulties maintaining accurate records across practices regarding consumer refusal to participate or those that were not offered a survey form; thus, there is no way to effectively calculate whether this was a representative sample  -Patients who were unable to make a timely appointment with the RN may have been underrepresented in the sample, which may skew results towards higher reported satisfaction |
| Halcomb, Salamonson, & Cook, 2015 | ICROMS Score: 17  Study Design: Observational; self-report survey  -Study does not contain information regarding the degree of autonomy/scope of practice that nurses had at each clinic  -Response rate was low (29.6%; data were only returned from 8/27 general practices who initially agreed to participate)  -The patient’s type and severity of chronic disease was not measured; it is possible that level of perceived health may impact patient satisfaction with the encounter  -Data gathered in study was entirely based on self-report  -Greater separation could have been achieved between the health providers and data collection (to minimize the social desirability effect)  -There was a substantial ceiling effect (63% of participants reported a maximum score), which may produce artificially skewed data  -Study contained a relatively small sample size (n=81) |
| Harris et al., 2015 | ICROMS Score: 28  Study Design: Cluster randomized controlled trial  -Study included a narrow age range (60-75 years) and may not be generalizable to a broader sample  -Participants in the intervention group were slightly older, less educated, more likely to be overweight/obese, more likely to have chronic diseases/disabilities, and had a slightly lower baseline step-count than those in the control group  -Neither the patients themselves or the researchers were blinded to the group allocation  -Some patients were randomized without meeting the eligibility cut-off for recorded activity (≥ 5 days of ≥ 600 minutes); additionally, 12 were incorrectly randomized  -Not all participants in the intervention group attended all four nurse counseling sessions  -Affluent, non-ethnically diverse socio-economic areas were overrepresented in the sample  -Intervention was comprised of multiple components, making it difficult to determine which components were contributing to outcomes  -Patients may have been more motivated to engage in physical activity given that they knew they were being monitored (i.e., Hawthorne effect); results may be less in “real world” scenarios |
| Harris et al., 2017 | ICROMS Score: 26  Study Design: Cluster randomized controlled trial  -Participants, nurses and researchers were unmasked to intervention allocation  -The RN-supported group had a slightly higher average daily step-count and minutes spent weekly in moderate to vigorous physical activity in bouts of ≥ 10 minutes at baseline than comparator groups  -Not all participants in the intervention group attended all three sessions  -Patients may have been more motivated to engage in physical activity given that they knew they were being monitored (i.e., Hawthorne effect); results may be less in “real world” scenarios  -Recruitment rate was low (10%), raising issues of generalizability  -At baseline, only 21% of patients achieved guidelines based on accelerometry but were not excluded |
| Iles et al., 2014 | ICROMS Score: 22  Study Design: Randomized controlled trial; cost-analysis  -There were small sample sizes for each chronic disease, as well as a slightly higher proportion of patients with diabetes in two of the general practices  -Study only contained 3 practice locations, which may limit generalizability  -Cost analysis is measured based on total MSB charges; however, this method tends to be somewhat limited in capturing the full extent of healthcare associated costs  -Difficult to determine what caused the increased workload during the intervention period, this cannot be attributed to quality of care per se  -There is no follow-up or long-term data to predict changes to cost-effectiveness over time  -It is unknown whether the additional costs would result in better long-term health outcomes |
| Karnon et al., 2013 | ICROMS Score: 22  Study Design: Observational; risk-adjusted cost-effectiveness analysis  -Study was specifically looking at the role of primary care RNs in the practice setting in general; level of RN involvement in the care of individual patients included in the study itself was not examined  -BMI was used as the main measurement, which is a somewhat flawed method of assessing body fat  -Study employed an observational design; there was no control group comparison or way to account for potential cofounders  -Uncertainty around cost-effectiveness, given that only 23% of all patients received inpatient care and the mean differences were driven by a few high-cost individuals  -Study would have benefitted from follow-up component to measure sustained differences over time  -Estimated quality-adjusted life year gain was based on the assumption that weight loss at 12 months would be regained over the following two years, which may not be the case  -Sample size was relatively small (n=175 across comparator groups)  -Study may have been subject to selection bias  -Differences were observed in the extent to which physicians recorded patient details (e.g. BMI) causing potential participants to be excluded and the observed effectiveness of nurse involvement in the high-level model to be underestimated  -There is some arbitrariness and uncertainty around the criteria used to allocate practices to the different models, as the categorization is based only on the RN’s subjective response. |
| Marshall et al., 2011 | ICROMS Score: 20  Study Design: Observational; cross-sectional survey  -Duration of intervention was not defined; results of first and last clinic attendance were compared, but the actual time duration between these two were not elaborated upon  -Study lacks details regarding specific recruitment measures and RN characteristics  -Response rate for consultation satisfaction survey was low (n=424/2850)  -There was an overall lack of significant changes in the clinical outcome measures, which may be a result of data collection issues (time span) or the type/accuracy of the outcomes measures  -The study target population of specific groups with known health inequalities (Maori, Pacific, males, and individuals from deprivation quintile 5) was not reached |
| Moher et al., 2001 | ICROMS Score: 26  Study Design: Pragmatic, unblinded cluster randomized controlled trial  -Primary care RNs received education on how to implement the specific clinic protocol and had ongoing support from the trial’s nurse facilitator; it is difficult to determine whether or not the same results would be generalizable to ‘usual’ primary care  -Information about antiplatelet treatment was confined to prescriptions, as the study did not collect records of patient self-medication  -The practices participating in the study tended to be large, with good pre-existing nursing support (as opposed to many of the non-participating practices that had minimal nursing support); therefore, results may not be generalizable  -Trial took place in the context of a health authority audit initiative relating to patients with coronary heart disease; the observed intervention effect may have been greater otherwise |
| O’Neill et al., 2014 | ICROMS Score: 18  Study Design: Observational; non-randomized retrospective comparison of a natural experiment  -RNs are involved in the team composition of both comparison groups; it is difficult to determine the extent to which outcomes can be specifically attributed to the RN role itself  -Intervention occurred over a very short time span (~1 month)  -There was a greater proportion of white patients in the physician group than the comparator  -Since only patients with hypertension were included, similar trends between both groups may have simply been a result of regression towards the mean  -Study is exploratory in design, therefore not designed to fully assess effectiveness  -The study setting (Veteran’s Affairs medical center) may not be generalizable to a standard patient population, given its primarily male patient population  -Study used previously documented, unstandardized data from a variety of clinicians; preventing the ability to control for confounding variables  -Patients who were non-adherent with treatment or follow-up were excluded from the analysis and information surrounding excluded patients was not collected, likely affecting outcomes |
| Pearson et al., 2003 | ICROMS Score: 22  Study Design: Uncontrolled before-after  -Secondary analysis of data  -Telephone follow-up was carried out by 12 RNs and 2 LPNs; there was no distinction between groups  -A psychiatrist worked closely with RNs and facilitated supervisory telephone sessions; difficult to determine if these results would be generalizable to RNs without this additional training/supervision  -The majority (80%) of the study population were women  -Study did not contain a control group to effectively evaluate the intervention  -Results of this particular study were analyzed prior to completion of the entire cohort (only two-thirds were included in the analysis); results may have changed  -Patients had a new diagnosis (or episode) of depression and were starting an SSRI treatment regimen; whether or not nurse intervention would have been effective for patients with varied depression histories and without medication was not determined  -If treatment with SSRIs failed, patients were prescribed other medications or were referred to psychiatric counseling, therefore introducing possible confounding variables |
| Pine et al., 1997 | ICROMS Score: 23; 24*  Study Design: Non-controlled before-and-after clinical trial followed by a non-randomized control trial  -Study involved only one practice location and 2 RNs  -Initial portion of study (pre-post study) did not involve a control group  -Patient sample was not ethnically diverse  -Eating habits were scored using a self-report questionnaire; it is possible that patients would embellish healthy habits and/or downplay negative eating habits (i.e., social desirability bias)  -Most of the participants (~70%) were not charged for visits, however some may have had to pay an office visit fee and a charge for follow-ups; patients who had to pay out-of-pocket may have been more motivated initially to change their dietary habits  -The intervention included only 5 sessions in the first study, and as few as 2 in the second study; it is possible that increased telephone follow-up may have improved dietary fat intake reduction.  -Most participants were already following a Step 1 Diet plan at the index visit; counseling may have had a larger effect on the general population (those not already diagnosed with high cholesterol)  -Reasons for non-participation was not addressed; study participants may not be representative of other patients with high cholesterol who refused referral to the intervention  -Study measured total cholesterol rather than low density lipoprotein levels (which is a much more robust measurement) |
| Waterfield et al., 2021 | ICROMS Score: 27  Study Design: Randomized controlled trial  -Participants in study were asymptomatic and attending the clinic for reasons unrelated to the intervention; it is difficult to assess whether outcomes would be generalizable to patients with more severe presenting symptoms  -Intervention was carried out between 2003-2005; research and guidance in this area has since been updated and findings may not be as relevant given the current evidence-base  -Attrition rate was somewhat high (34%); however, this is consistent with the rate for studies aimed at health promotion/prevention rather than symptom control/treatment  -Recruitment continued throughout study as a means of accounting for drop-outs; this may result in the study being ‘overpowered’  -The trial was not blinded, as it is not possible to provide blinding in studies where participants have to adhere to a particular form of exercise. Likewise, it was not possible to blind the RN or urogynecology nurse specialist who was providing the intervention. However, the use of an objectively measured primary outcome may mitigate this  -Adherence to the pelvic floor exercise regimen was based on participant report; the extent to whether or not they accurately followed the recommendations is not verifiable |
| Zwar et al., 2010 | ICROMS Score: 22  Study Design: Non controlled pre- and post-study using mixed methods  -RNs received substantial mentoring and support from a project officer and the role was supported by an ongoing source of funding; results may not be generalizable outside of these circumstances  -Intervention duration was very brief (4 weeks)  -Eligibility criteria for practices simply required that there be one or more RNs employed; level of autonomy/scope of practice, types of roles, etc., were not taken into consideration or explored  -Smoking abstinence 7 days prior to assessment was only based on patient self-report  -Purpose of physician visits were not recorded; these could have been for reasons other than smoking cessation counseling, making it difficult to draw comparisons  -Participants lost to follow-up were classified as continuing smokers by default  -Due to difficulties with the availability of the measurement tool, expired CO2 validation of smoking status was not carried out in a substantial number of patients  -Study looked at 12 month anticipated relapse rate based on external data; an actual follow-up of patients would have yielded more reliable information  -Study did not use a control group  -Intervention involved both RN counseling and the use of NRTs, making it difficult to differentiate between the effects of each component |
| **Mixed methods study consisting of multiple designs; separate ICROMS quality appraisal scores were generated for each study type; RN – registered nurse; NRT – nicotine replacement therapy; HbA1C – hemoglobin A1C; MSB – Medicare Benefits Schedule; BMI – body mass index; LPN – licensed practice nurse; SSRI – selective serotonin reuptake inhibitor; CO2 – carbon dioxide* | |
